# 4-(3-Phenyl-4-(3,4,5-trimethoxybenzoyl)-1*H*-pyrrol-1-yl)benzenesulfonamide, a Novel Carbonic Anhydrase and Wnt/β-Catenin Signaling Pathway Dual-Targeting Inhibitor with Potent Activity against Multidrug Resistant Cancer Cells

**DOI:** 10.1021/acs.jmedchem.3c01424

**Published:** 2023-10-30

**Authors:** Domiziana Masci, Michela Puxeddu, Laura Di Magno, Michele D’Ambrosio, Anastasia Parisi, Marianna Nalli, Ruoli Bai, Antonio Coluccia, Pietro Sciò, Viviana Orlando, Sara D’Angelo, Stefano Biagioni, Andrea Urbani, Ernest Hamel, Alessio Nocentini, Serena Filiberti, Marta Turati, Roberto Ronca, Joanna Kopecka, Chiara Riganti, Cinzia Fionda, Rosa Bordone, Giorgia Della Rocca, Gianluca Canettieri, Claudiu T. Supuran, Romano Silvestri, Giuseppe La Regina

**Affiliations:** † Department of Basic Biotechnological Sciences, Intensivological and Perioperative Clinics, Catholic University of the Sacred Heart, Largo Francesco Vito 1, Rome 00168, Italy; ‡ Laboratory Affiliated with the Institute Pasteur Italy—Cenci Bolognetti Foundation, Department of Drug Chemistry and Technologies, Sapienza University of Rome, Piazzale Aldo Moro 5, Roma 00185, Italy; § Laboratory Affiliated to Istituto Pasteur Italia—Fondazione Cenci Bolognetti, Department of Molecular Medicine, Sapienza University of Rome, Viale Regina Elena 291, Rome 00161, Italy; ∥ Molecular Pharmacology Branch, Developmental Therapeutics Program, Division of Cancer Treatment and Diagnosis, Frederick National Laboratory for Cancer Research, National Cancer Institute, National Institutes of Health, Frederick, Maryland 21702, United States; ⊥ Department of Biology and Biotechnologies “Charles Darwin”, Sapienza University of Rome, Piazzale Aldo Moro 5, Rome 00185, Italy; # Dipartimento Neurofarba, Sezione di Scienze Farmaceutiche e Nutraceutiche, Universitá degli Studi di Firenze, Via Ugo Schiff 6, Sesto Fiorentino I-50019, Firenze, Italy; ¶ Experimental Oncology and Immunology Unit, Department of Molecular and Translational Medicine, University of Brescia, Via Branze 39, Brescia 25123, Italy; ∇ Department of Oncology and Molecular Biotecnology Center “Guido Tarone″, Oncological Pharmacology Unit, Via Nizza 44, Torino 10126, Italy

## Abstract

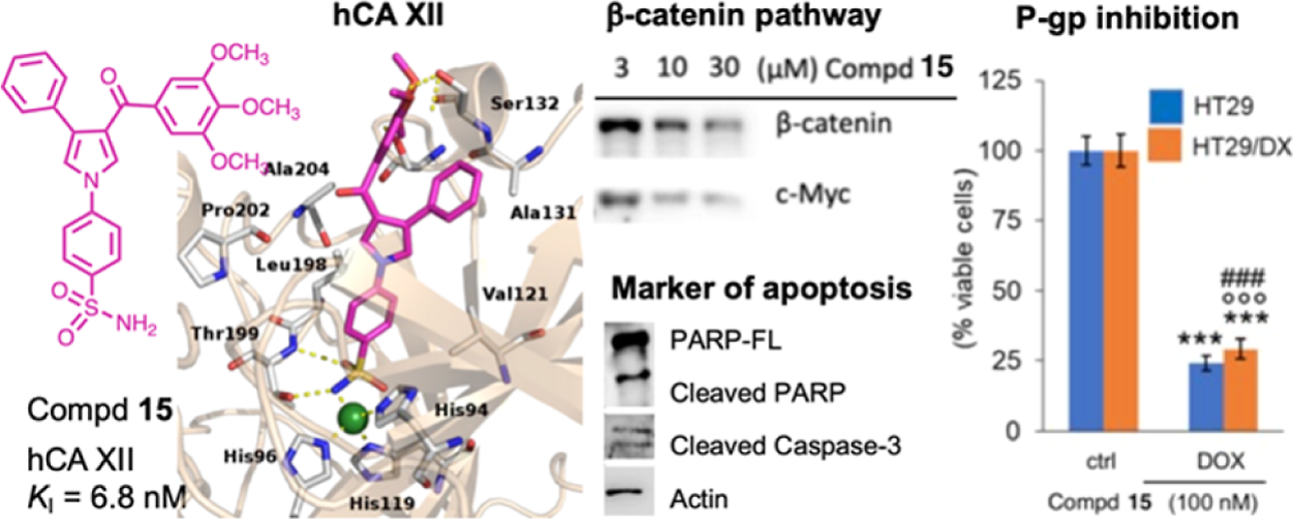

We synthesized new pyrrole and indole derivatives as human carbonic anhydrase (hCA) inhibitors with the potential to inhibit the Wnt/β-catenin signaling pathway. The presence of both *N*1-(4-sulfonamidophenyl) and 3-(3,4,5-trimethoxyphenyl) substituents was essential for strong hCA inhibitors. The most potent hCA XII inhibitor **15** (*K*_i_ = 6.8 nM) suppressed the Wnt/β-catenin signaling pathway and its target genes MYC, Fgf20, and Sall4 and exhibited the typical markers of apoptosis, cleaved poly(ADP-ribose)polymerase, and cleaved caspase-3. Compound **15** showed strong inhibition of viability in a panel of cancer cells, including colorectal cancer and triple-negative breast cancer cells, was effective against the NCI/ADR-RES DOX-resistant cell line, and restored the sensitivity to doxorubicin (DOX) in HT29/DX and MDCK/P-gp cells. Compound **15** is a novel dual-targeting compound with activity against hCA and Wnt/β-catenin. It thus has a broad targeting spectrum and is an anticancer agent with specific potential in P-glycoprotein overexpressing cell lines.

## Introduction

Carbonic anhydrases (CAs, EC 4.2.1.1) are ubiquitous enzymes that catalyze the reversible hydration of carbon dioxide to produce monohydrogen carbonate and H^+^ ions.^1^ CAs fall into eight different classes, α- to ι-CAs, but only the α-class is found in mammals. Several genetically distinct CA isoforms in the α-class family, including the human CA (hCA) isoforms, have been recognized.^2,3^ They are cytosolic proteins, mitochondrial matrix proteins, transmembrane proteins, or proteins linked by means of glycosylphosphatidylinositol tails to the plasma membrane. The CA isoforms play key roles in many physiological processes, such as pH homeostasis, electrolyte secretion, biosynthesis of several metabolites, signal transduction, cell differentiation and proliferation, and oncogenesis.^4,5^

Dysregulated expression and/or abnormal activity of specific CA isoforms cause onset of several pathological conditions.^6^ The cytosolic CAs I and II are isoforms ubiquitously spread throughout the human body. The CA II plays a relevant physiological role and is found highly expressed not only in red blood cells, the gastrointestinal tract, the lungs, and the kidneys^7^ but also in some types of cancer, such as urothelial carcinoma.^8^ Both isoforms are involved in glaucoma,^9^ epilepsy,^10^ and Parkinson’s disease.^11^ The human-membrane-associated CA IX and CA XII enzymes catalyze the hydration of carbon dioxide in the extracellular space. Overexpression of both the CA IX and CA XII isoforms is triggered by the hypoxia-inducible factor 1 (HIF-1) in many types of cancer.^12^ CA IX is expressed in breast carcinoma, colorectal cancer (CRC), glioblastoma, lung cancer, and cervical squamous cell carcinoma.^13^ High levels of CA XII have been detected in breast cancer,^14^ nonsmall cell lung cancer,^15^ cervical cancer^16^ and gliomas, hemangioblastomas, and meningiomas.^17^ A spliced form of CA XII is expressed in brain tumors.^18^ Cystic fibrosis-like syndrome and hyponatremia have been associated with a mutation in CA XII.^19^ In CRC, the expression of the CA II and CA XII isoforms has been correlated with patient survival, in that higher expression indicated poorer prognosis, and this suggested their potential role as prognostic biomarkers.^20^

**Figure 1:**
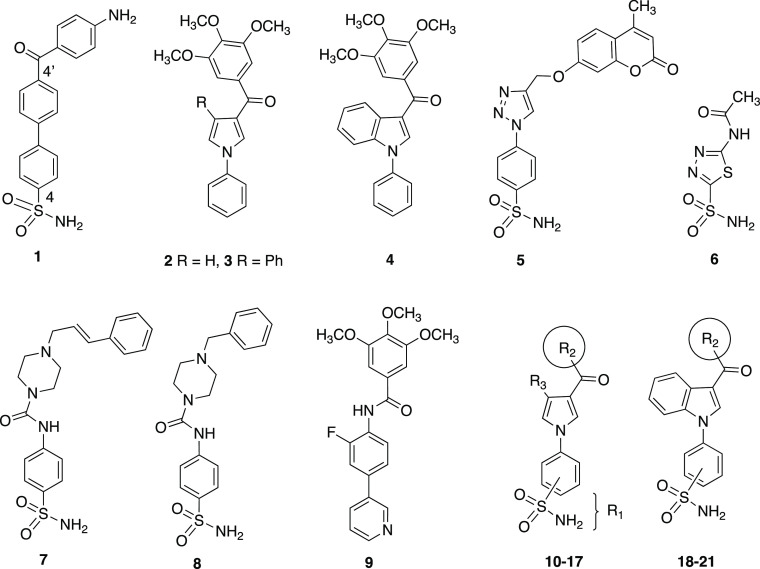
Structures **1–21**. Compounds: **1**, CA inhibitor; **2**–**5**, tubulin polymerization inhibitors; **6**, acetazolamide; **7,8**: Wnt/β-catenin modulators; **9**: P-gp modulator; and **10**–**21**: planned inhibitors (see Table 1 for R_1_–R_3_ substituents).

In our previous studies, we synthesized 1,1′-biphenylsulfonamides as potent inhibitors of the hCA isoforms, with nM *K*_i_ values.^21,22^ The biphenylsulfonamide **1** showed strong inhibition of hCA isoforms XII and XIV. In this work, we designed new CA inhibitors by introducing a sulfonamide group at the *N*1-phenyl of **2**–**4**,^23−25^ whose primary activity was the inhibition of tubulin polymerization, to give compounds **10**–**21** (Figure 1 and Table 1). Compounds **11**, **15**, and **19** showed strong inhibition of hCA isoforms I, II, IX, and XII with low nM *K*_I_ values (Table 1). These compounds did not significantly inhibit tubulin polymerization, although sulfonamide **5** was reported by Guo^26^ to inhibit tubulin assembly. Compound **11** did not inhibit tubulin assembly at 20 μM, and **15** and **19** showed only partial inhibition at 20 μM (in this assay, combretastatin A-4 inhibited the tubulin assembly with an IC_50_ of 0.75 μM) (Table S1, Supporting Information). Overexpression of CA II and β-catenin signaling in urothelial carcinoma was reported by Matsue.^27^ Although the mechanism was not fully elucidated, the authors hypothesized that CA II may induce epithelial–mesenchymal transition (EMT) upregulating Wnt/β-catenin signaling.^28^ Reference drug acetazolamide **6** (AAZ) was demonstrated to suppress Wnt/β-catenin signaling.^29^ Sulfonamides **7** and **8** were reported by Fang to inhibit Wnt signaling by interfering with both β-catenin/Tcf4 and β-catenin/LEF1 in protein extracts from HCT116 cells.^30^ The CA XII isoform is required for optimal activity of the P-glycoprotein (P-gp). Increasing CA XII levels at the plasma membrane of P-gp positive cancer cells were found during the acquisition of chemoresistance.^31^ P-gp-mediated chemoresistance is reversed by CA XII inhibitors.^32^ The 3,4,5-trimethoxybenzoyl moiety of compound **9** was required for modulating P-gp levels.^33^

**Table 1:**
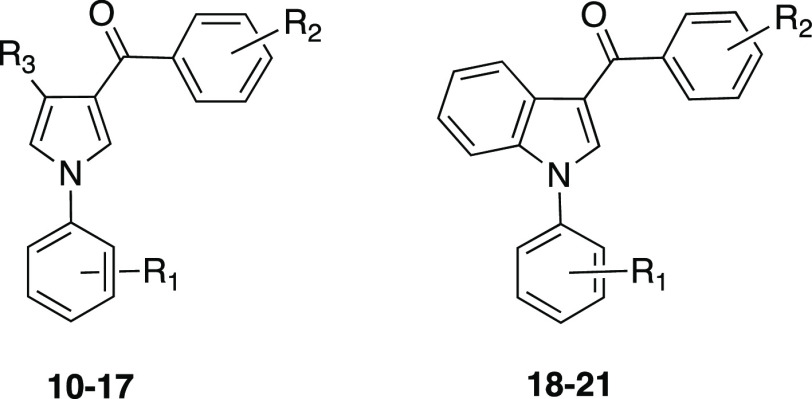
Inhibition Data of Human CA Isoforms I, II, IX, and XII by Compounds 10–21 and Reference Compounds **1** and AAZ^c^

				*K*_i_ (nM)^a^^,^^b^			
compd	R_1_	R_2_	R_3_	hCA I	hCA II	hCA IX	hCA XII
**10**	4-SO_2_NH_2_	H	H	73.1	57.5	66.0	47.1
**11**	4-SO_2_NH_2_	3,4,5-(OCH_3_)_3_	H	95.6	10.2	44.8	11.5
**12**	3-SO_2_NH_2_	3,4,5-(OCH_3_)_3_	H	643.8	84.2	34.7	21.3
**13**	2-SO_2_NH_2_	3,4,5-(OCH_3_)_3_	H	5837	887.5	228.6	425.4
**14**	4-SO_2_NH_2_	H	phenyl	208.9	72.4	38.6	59.4
**15**	4-SO_2_NH_2_	3,4,5-(OCH_3_)_3_	phenyl	272.1	33.6	24.1	6.8
**16**	3-SO_2_NH_2_	3,4,5-(OCH_3_)_3_	phenyl	1028	186.5	39.1	28.9
**17**	2-SO_2_NH_2_	3,4,5-(OCH_3_)_3_	phenyl	>10,000	>10,000	8863	1003
**18**	4-SO_2_NH_2_	H		327.8	41.7	31.6	52.0
**19**	4-SO_2_NH_2_	3,4,5-(OCH_3_)_3_		446.4	67.8	7.3	16.5
**20**	3-SO_2_NH_2_	3,4,5-(OCH_3_)_3_		942.8	228.9	69.0	58.1
**21**	2-SO_2_NH_2_	3,4,5-(OCH_3_)_3_		>10,000	>10,000	7751	1193
**1**				62.0	86.2	9.2	25.4
AAZ^d^				250.0	12.0	25.0	5.7

a Mean from three different assays, by a stopped flow technique.

b Standard deviations were in the range of ±5–10% of the reported *K*_i_ values.

c No data.

d AAZ, acetazolamide.

**Figure 2:**
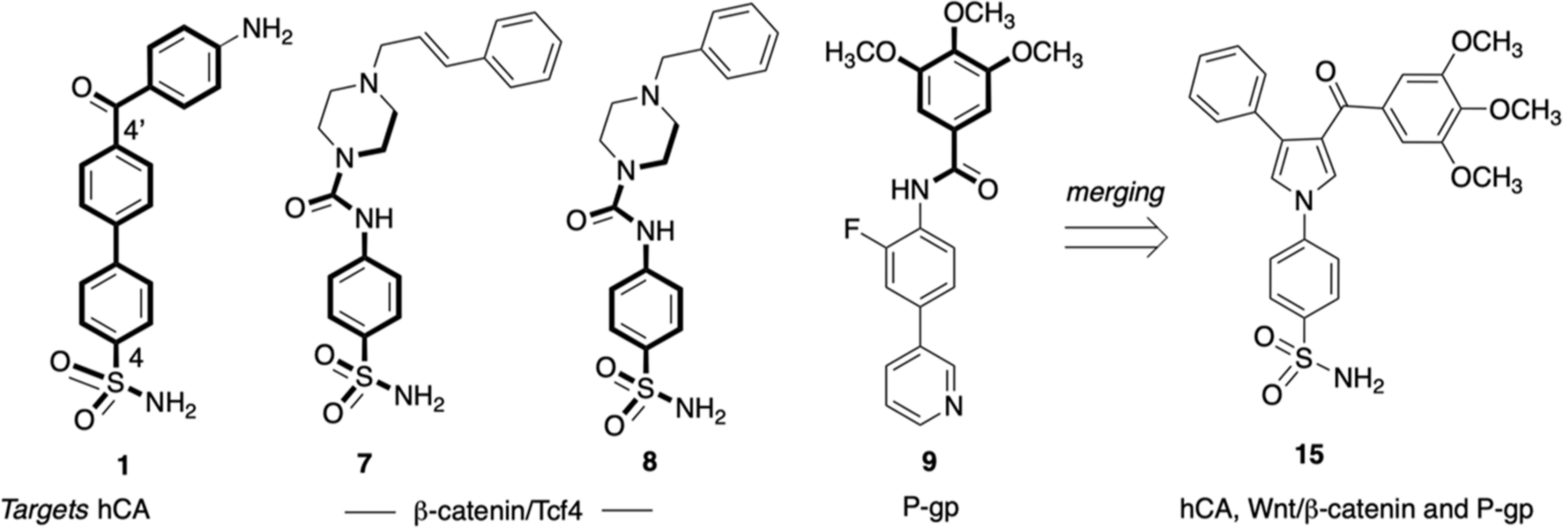
Structural elements that have been merged to achieve the multitarget activity of **15**.

Compound **15**, the most potent hCA XII inhibitor within the series, merges key structural features present in compounds **1**, **7**, **8**, and **9**, namely, the 4′-benzoyl-(1,1′-biphenyl)-4-sulfonamide of **1**, the 4-ureidobenzenesulfonamide of **7** and **8**, and the 3,4,5-trimethoxybenzoyl moiety of **15**. A summary sketch of the elements that have been merged to achieve the multitarget activity of compound **15** is shown in Figure 2. We were intrigued by the ability of compound **15** to interfere with the Wnt/β-catenin pathway and to restore the drug sensitivity of P-gp-overexpressing cancer cells. Indeed, **15** inhibited effectively the Wnt/β-catenin pathway and its target gene MYC, and besides its activity in NCI/ADR-RES cells, restored the sensitivity to doxorubicin (DOX) in HT29/DX and MDCK/P-gp cells. In the HT29/DX model, **15** at 100 nM in combination with DOX decreased the cell viability obtained with DOX alone in these chemosensitive cells.

## Results and Discussion

### Chemistry

**Scheme 1:**
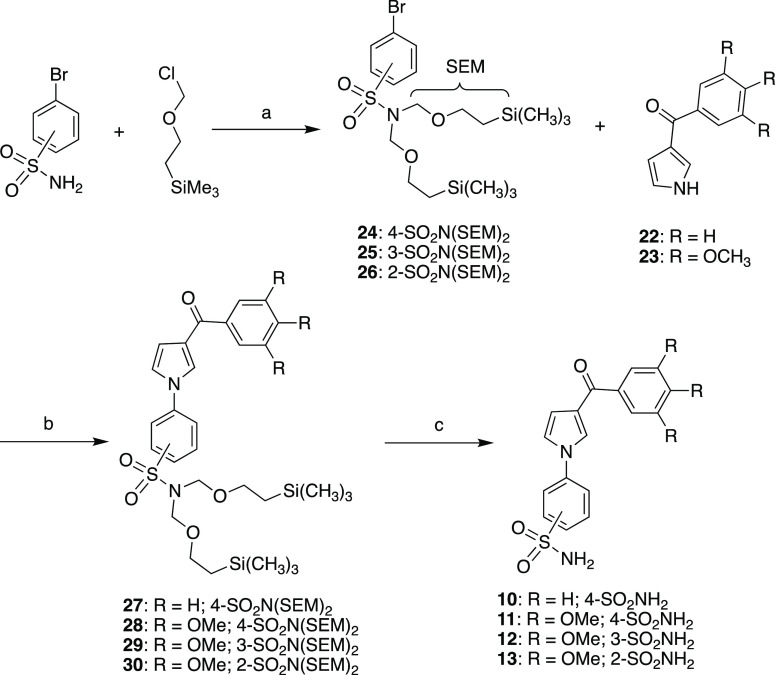
Synthesis of Sulfomanides **10–13** (a) NaH, DMF, RT, 50 min, Ar, 90–98%; (b) CuI, Cs_2_CO_3_, 1,10-phenanthroline, 1,4-dioxane, MW, closed vessel, 210 °C, 200 W, 40 min, 17–61%; (c) TBAF, THF, reflux, 4 h, 70–90%.

Sulfonamides **10**–**13** were prepared as depicted in Scheme 1. 3-Aroylpyrroles **10** and **11** were synthesized starting by reaction of ketones **22** or **23**(34) with the 2-(trimethylsilyl)ethoxymethyl (SEM) protected 4-bromo-*N*,*N*-bis((2-(trimethylsilyl)ethoxy)methyl)benzenesulfonamide (**24**) in the presence of copper(I) iodide, cesium carbonate, and 1,10-phenanthroline under microwave irradiation at 210 °C, 200 W for 40 min to give 4-(3-benzoyl-1*H*-pyrrol-1-yl)-*N*,*N*-bis((2-(trimethylsilyl)ethoxy)methyl)-benzenesulfonamide (**27**) or the corresponding 3-(3′,4′,5′-trimethoxybenzoyl-1*H*-pyrrol-1-yl) derivative (**28**). Similarly, **29** and **30** were obtained starting from **23** and 3-bromo-*N,N*-bis((2-(trimethylsilyl)ethoxy)methyl)benzenesulfonamide (**25**) or 2-bromo-*N,N*-bis((2-(trimethylsilyl)ethoxy)methyl)benzenesulfonamide (**26**), respectively. Finally, the SEM protecting groups of **27**–**30** were removed by heating under reflux in tetrahydrofuran (THF) for 4 h in the presence of tetrabutylammonium fluoride (TBAF). The SEM-protected bromophenylsulfonamides **24**–**26** were obtained by reaction of 4-brombenzenesulfonamide, or its 2- or 3-bromo counterparts, with trimethylsilinethoxymethyl chloride in the presence of sodium hydride in *N,N*-dimethylformamide at room temperature for 50 min under an argon stream (Scheme 1).

**Scheme 2:**
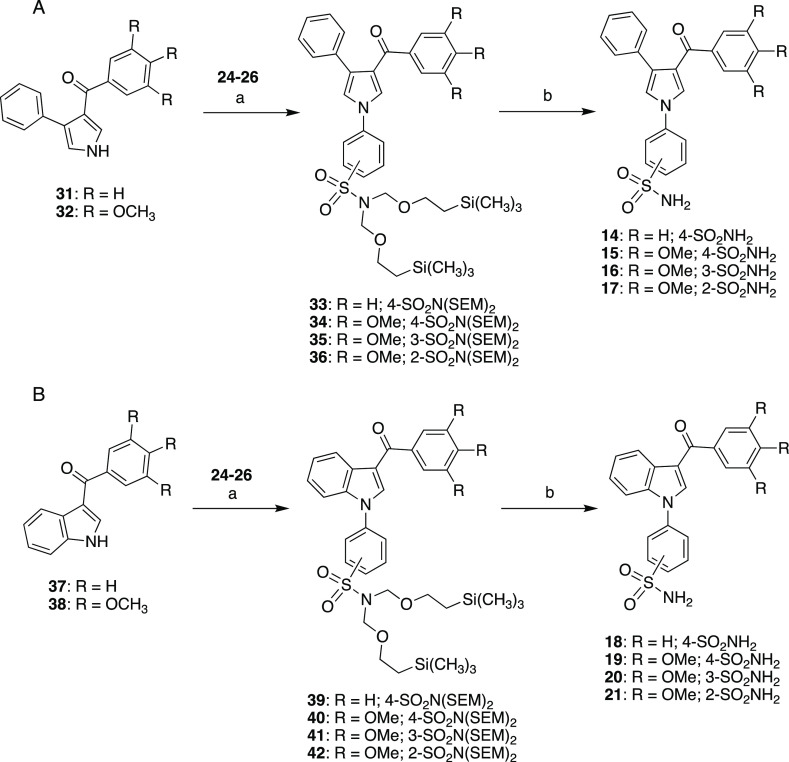
Synthesis of Sulfonamides **14-17** (Top Panel) and **18-21** (Bottom Panel) (a) CuI, Cs_2_CO_3_, 1,10-phenanthroline, 1,4-dioxane, MW, closed vessel, 210 °C, 200 W, 40 min, 24–65%; (b) TBAF, THF, reflux, 4 h, 71–98%.

Sulfonamides **14**–**21** were prepared by similar routes, as depicted in Scheme 2. Compounds **14**–**17** were synthesized starting from 3-aroyl-4-phenylpyrroles **31**(35) and **32**(34) (Scheme 2, panel A) and **18**–**21** starting from 3-aroylindoles **37** and **38**(36) (Scheme 2, panel B).

### Biology

#### Inhibition of hCA I, hCA II, hCA IX, and hCA XII Isoforms

**Figure 3:**
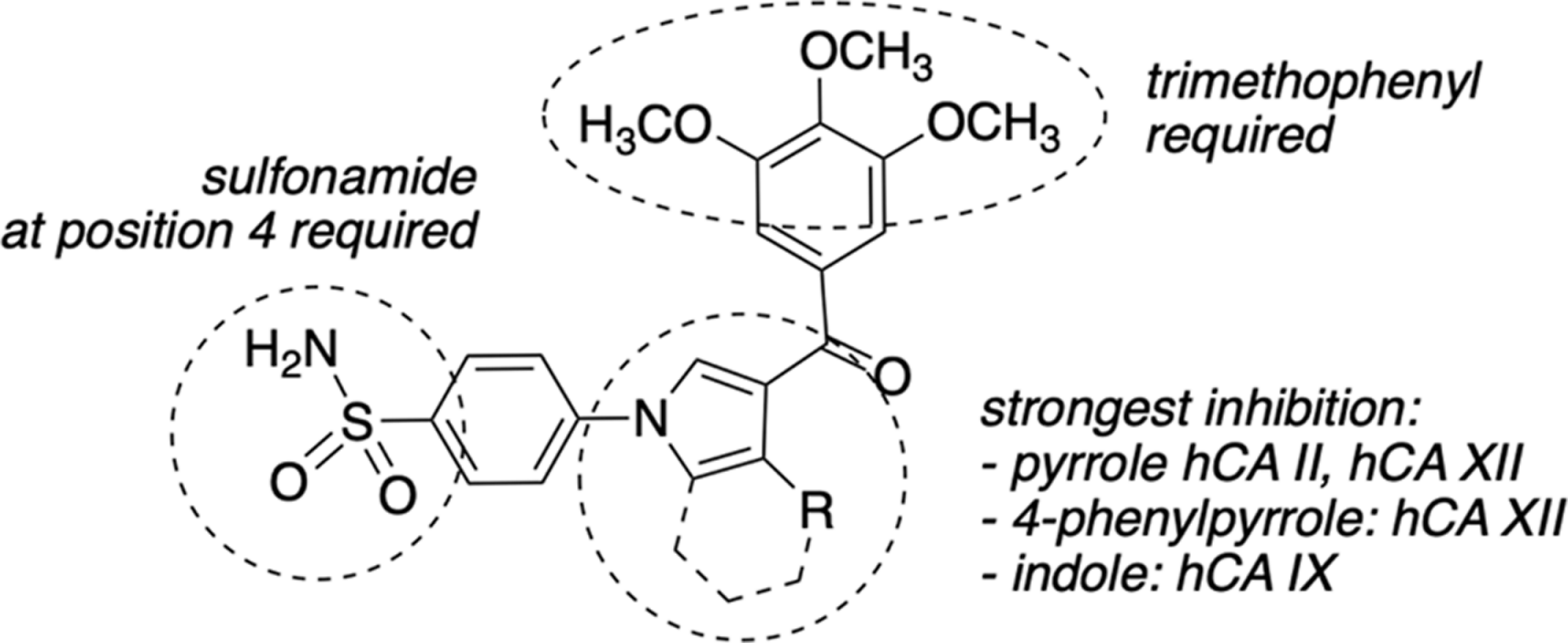
SAR summary of hCA I, hCA II, hCA IX, and hCA XII inhibition by compounds **10**–**21**.

Inhibition data of sulfonamides **10**–**21** against human isoforms CA I, II, IX, and XII were measured by a stopped flow CO_2_ hydrase assay.^37^ The *K*_i_ values of **10**–**21** are presented in Table 1, along with that of the reference drug AAZ, a CA inhibitor medication approved by the FDA to treat glaucoma, idiopathic intracranial hypertension, congestive heart failure, altitude sickness, periodic paralysis and epilepsy,^38^ and of the previously reported 4,4′-biphenylsulfonamide **1**.^21^ Structure–activity relationships (SARs) can be gathered from the *K*_i_ inhibition values reported in Table 1. As inhibitors of hCA I, pyrrole derivatives **10** (*K*_i_ = 73.1 nM) and **11** (*K*_i_ = 95.6 nM) were more potent than the corresponding 4-phenylpyrroles **14** and **15** and indoles **18** and **19** and were at the same level of the reference 1,1′-biphenylsulfonamide **1** (*K*_i_ = 62.0 nM). Shifting the sulfonamide moiety from the *para* position in **11** to *meta* or *ortho* position in **12** or **13** resulted in a 6.7- and 61.0-fold reduction in the inhibition of hCA I, respectively. Sulfonamides **11**, **15**, and **18** yielded hCA II inhibition with *K*_i_ values < 50 nM. In particular, compound **11** showed the strongest inhibition of hCA II (*K*_i_ = 10.2 nM) within the series and was comparable to AAZ (*K*_i_ = 12.0 nM). Several sulfonamides inhibited hCA IX with *K*_i_ < 50 nM. Compound **15** (*K*_i_ = 24.1 nM) was equipotent to AAZ (*K*_i_ = 25.0 nM). The most potent hCA IX inhibitor **19** (*K*_i_ = 7.3 nM) was superior to the references **1** (*K*_i_ = 9.2 nM) and AAZ (*K*_i_ = 25.0 nM). Sulfonamides, **10**–**12**, **15**, **16**, and **19**, inhibited hCAXII with *K*_i_ < 50 nM. In all series, the strong inhibition correlated with the presence of the sulfonamide at the *para* position and with the 3,4,5-trimethoxyphenyl group. Pyrrole derivatives **11** (*K*_i_ = 11.5 nM) and **15** (*K*_i_ = 6.8 nM) showed the strongest hCA XII inhibition. As inhibitors of hCA XII, **11** and **15** were superior to the reference compound **1**, and **15** was comparable to AAZ. **A** SAR summary of inhibition of hCA I, hCA II, hCA IX, and hCA XII by compounds **10**–**21** is depicted in Figure 3.

#### Molecular Modeling on hCA I, hCA II, hCA IX, and hCA XII Isoforms

Docking experiments were performed to gain insights into the molecular details of the binding modes of the reported compounds. Representative structures for each studied hCA isoform were selected from the protein data bank (PDB) server. The compounds were docked by Vina to the catalytic site of the enzymes. Inspection of the proposed docking binding conformations revealed the crucial interaction between the sulfonamide nitrogen atom and the catalytic zinc atom. The position *para*, *meta*, or *ortho* of the sulfonamide at the 1-phenyl ring markedly affected the binding interaction; the most active compounds all had the sulfonamide at the *para* position. This resulted in its contact involving the zinc atom of the enzyme and the sulfonamide moiety formed an H-bond between an oxygen atom and the Thr199 residue. The phenyl ring bearing the sulfonamide formed hydrophobic interactions with Leu198, a residue that was conserved in all the studied isoforms, and Val121 which became an Ala residue in hCA I. When the sulfonamide group was at the *meta* or *ortho* position, a steric clash resulted in attempting to achieve a proper bond distance from the zinc atom.

**Figure 4:**
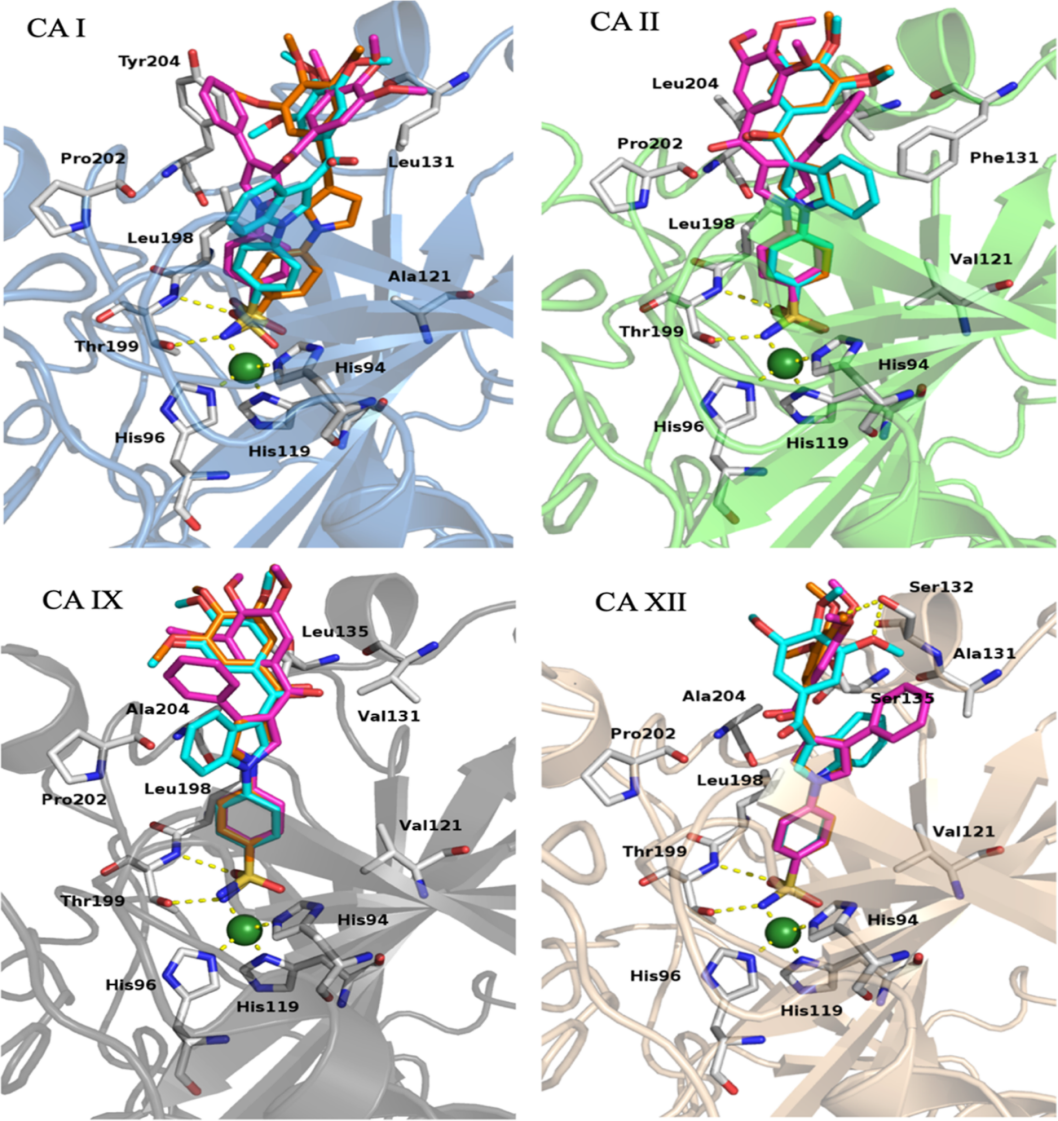
Proposed binding mode of derivatives **11** (orange), **15** (magenta), and **19** (cyan). Enzymes are shown as a colored cartoon: hCA I, light blue; hCA II, green; hCA IX, gray; and hCA XII, sand. The zinc atom is depicted as a green sphere; residues involved in interactions are shown as white sticks; H-bonds are depicted as yellow dot lines. For the sake of clarity, amino acid residue numbers refer to hCA II.

Inspection of the binding modes led to the identification of hydrophobic interactions of the central pyrrole or indole ring with Pro202 and Leu198, both of which are conserved in all of the studied isoforms. Other hydrophobic interactions involved residues Leu131 in hCA I, Phe131 in hCA II, Val131, and Ala131 in hCA IX and Ala131 hCA XII. The 3,4,5-trimethoxyphenyl formed hydrophobic interactions with Phe131, Val135, and Leu204 in the case of hCA II and with Val131, Leu135, and Ala204 in the case of hCA IX. For hCA XII, along with the hydrophobic stabilization provided by the trimethoxyphenyl, an H-bond interaction between Ser132 and a methoxy group was observed. In the case of hCA I, the presence of the bulkier Tyr204 residue instead of Leu (hCA II), Ala (hCA IX), or Asn (hCA XII) forced the trimethoxyphenyl moiety into a different binding conformation that caused lower-quality binding. The binding modes provided by the docking experiments were consistent with the biological data and led to the identification of crucial structural requirements for inhibition of the enzyme (Figure 4).

#### Inhibition of Tubulin Polymerization and Docking in the Colchicine Site

At 20 μM, compound **11** did not inhibit tubulin assembly, whereas **15** and **19** resulted in partial inhibition. In the same assay, combretastatin A-4 (CSA4) as a reference compound inhibited the assembly reaction with an IC_50_ of 0.75 μM and colchicine binding with 99% inhibition. For comparison, **2**, the parent compound of **11**, inhibited tubulin assembly with an IC_50_ of 1.5 μM and colchicine binding by 81%;^23^**3**, the parent compound of **15**, inhibited tubulin assembly with IC_50_ of 1.1 μM and colchicine binding by 66%.^24^ Docking studies of derivatives **11**, **15**, and **19** at the colchicine site of the tubulin dimer highlighted that binding modes of **11** and **15** were consistent with those of the previously reported parent compounds **2**(23) and **3**,^24^ respectively; however, the sulfonamide group had a negative effect on binding to the colchicine site. Inspection of the docking poses revealed that while the sulfonamide groups of **11**, **15**, and **19** could fit into a hydrophobic pocket, there were no stabilizing contacts. This may suggest that the desolvation energy cost was not balanced by the binding energy of the sulfonamide moiety.^39^ For all derivatives, we hypothesized that the binding fitness got worse upon the introduction of the sulfonamide group mainly due to electronic rather than steric reasons (Figure S1, Supporting Information).

#### Inhibition of Growth of HCT-116, SW480, and SW620 Cancer Cells

Compounds **11**, **15**, and **19** were evaluated as inhibitors of the growth of the HCT116, SW480, and SW620 colorectal carcinoma cells (Table 2 and Figure S2, Supporting Information). All cell lines are characterized by enhanced activity of the Wnt/β-catenin pathway due to the CTNNB1 mutated gene present in the HCT116 cells that encodes β-catenin^40^ and mutation in the APC tumor suppressor gene in SW480 and SW620 cells.^41,42^ Compounds **15** and **19** inhibited the HCT116 cells with IC_50_’s of 8.7 and 14.9 μM, respectively, compared with 5-fluorouracil (5-FU) (IC_50_ = 8.2 μM). Both SW480 and SW620 cell lines were less sensitive to **15** and **19** than the HCT116 cells, with IC_50_ values ranging from 17.7 μM (**15**, SW480 cells) to 45.4 μM (**19**, SW620 cells). However, it should be noted that **15** and **19** were remarkably more potent than 5-FU as inhibitors of both the SW480 and SW620 cell lines. As an inhibitor of the SW480 and SW620 cells, 5-FU yielded IC_50_’s of 217.5 and 102.6 μM, respectively, and was 27- and 13-fold less effective than that in the HCT116 cells. In drug combination studies, each compound was used at the corresponding IC_50_ concentration. The combination **15** + 5-FU inhibited the HCT116 cells with an IC_50_ of 17.0 μM, two-fold higher than **15** as a single agent, while **19** + 5-FU was at the same level as **19** alone. As inhibitors of the SW480 and SW620 cells, **15** + 5-FU and **19** + 5-FU combinations were superior to 5-FU as a single agent; in the SW620 cells, **19** + 5-FU was 1.8-fold superior than **19** alone.

**Table 2:** Inhibition of Growth of HCT-116, SW480, and SW620 Cancer Cells by Compounds **15** and **19**^a^

	IC_50_ (μM)		
compd	HCT116^b^	SW480^b^	SW620^b^
**15**	8.7 ± 1.4	17.7 ± 1.3	23.8 ± 1.4
**19**	14.9 ± 1.3	37.4 ± 1.4	45.4 ± 1.7
5-FU	8.2 ± 1.5	217.5 ± 1.3	102.6 ± 2.4
**15** + 5-FU^c^	17.0 ± 8.0	42.9 ± 1.2	37.3 ± 1.2
**19** + 5-FU^c^	14.0 ± 2.1	50.3 ± 1.2	25.8 ± 1.2

a Experiments were performed in duplicate or triplicate.

b Human colorectal carcinoma cells.

c **15**, **19**, and 5-FU were at the corresponding IC_50_ concentrations.

#### Inhibition of Growth of MCF-7, NCI/ADR-RES, and OVCAR-8 Cancer Cells

Compounds **11**, **15**, and **19** were evaluated as inhibitors the growth of human nonmetastatic breast cancer epithelial cells (MCF-7), the ovarian tumor cell line 8 (OVCAR-8), and its derived NCI/ADR-RES DOX-resistant cell line that overexpresses P-gp, resulting in the type 1 multidrug-resistance phenotype (Table 3). Compounds **11**, **15**, and **19** inhibited the MCF-7, OVCAR-8, and NCI/ADR-RES cancer cells with IC_50_ values ranging from 1.9 μM (**15**, MCF-7 cells; **11** and **15**, NCI/ADR-RES and OVCAR-8 cells) to 4.8 (**19**, OVCAR-8 cells). Compound **15** was almost uniformly active against these three cell lines in the 1.9–2.1 μM range. Noteworthy, compounds **1**, **12**, and **19** were equipotent as inhibitors of the NCI/ADR-RES DOX-resistant cell line (EC_50_’s of 2.1–2.2 μM), uniformly more active than the tubulin assembly inhibitor vinorelbine (IC_50_ = 5.0 ± 1.0 μM) and slightly less potent than microtubule-stabilizing agent paclitaxel (IC_50_ = 1.5 ± 0.7 μM).^43^

**Table 3:** Inhibition of Growth of MCF-7, OVCAR-8, and NCI/ADR-RES Cell Lines by Compounds **11**, **15**, and **19**^a^

	IC_50_ (μM)		
compd	MCF-7^b^^,^^c^	OVCAR-8^d^	NCI/ADR-RES^e^
**11**	3.9 ± 0.08	1.9 ± 0.07	2.2 ± 0.5
**15**	1.9 ± 0.05	1.9 ± 0.07	2.1 ± 0.6
**19**	3.6 ± 1.0	4.8 ± 0.3	2.1 ± 0.2

a Experiments were performed in duplicate or triplicate.

b MCF-7 human nonmetastatic breast cancer epithelial cells.

c In this assay, CSA4 as a reference compound yielded IC_50_ of 15 ± 4 nM.

d OVCAR-8: ovarian tumor cell line 8.

e NCI/ADR-RES: DOX-resistant cell line derived from OVCAR-8.

#### Inhibition of TNBC Cancer Cells

**Figure 5:**
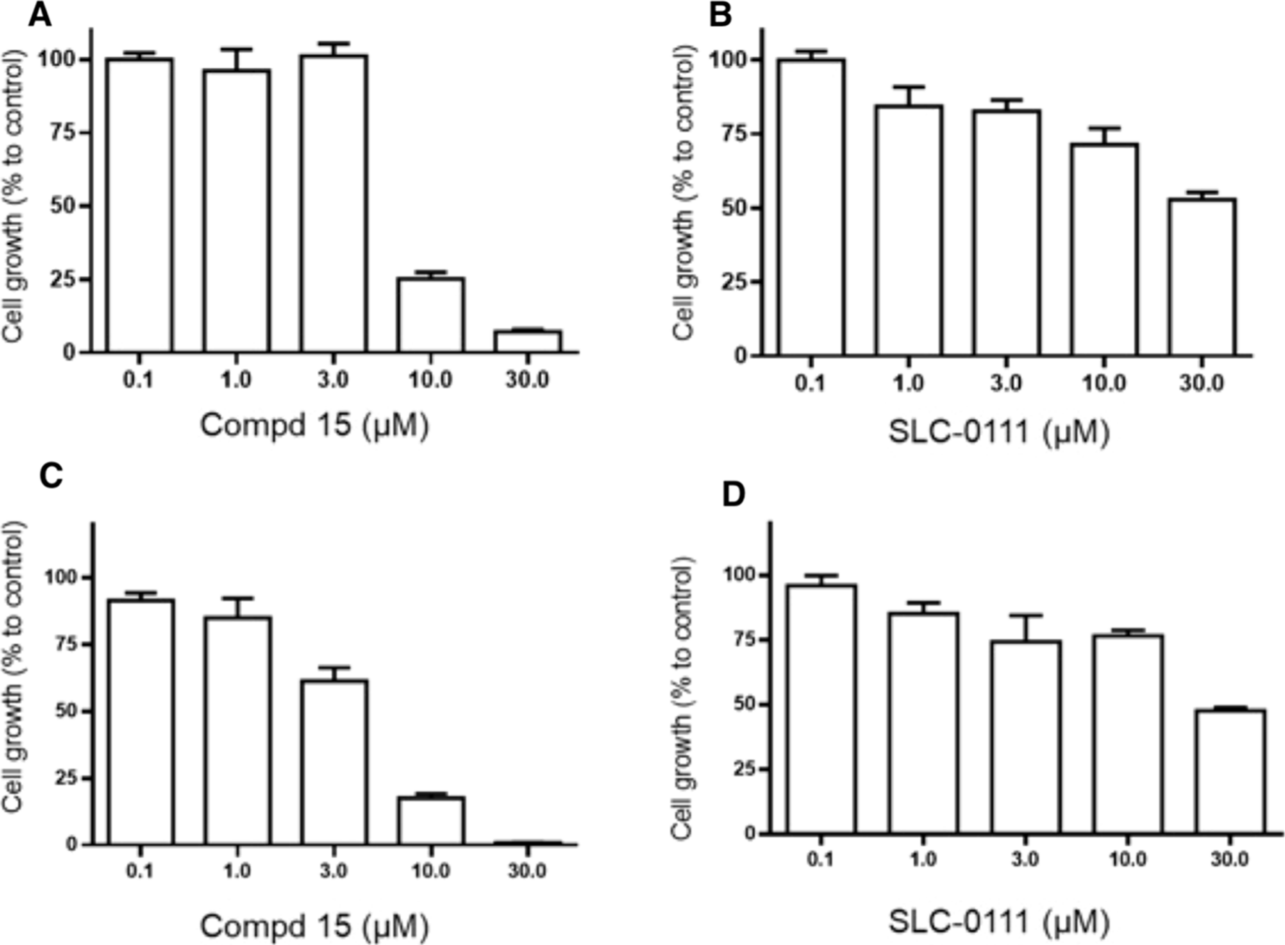
(A) Inhibition of MDA-MB 231 TNBC cell growth by compound **15**. (B) Inhibition of MDA-MB 231 TNBC cell growth by reference compound SLC-0111. (C) Inhibition of BT-549 TNBC cell growth by compound **15**. (D) Inhibition of BT-549 TNBC cell growth by reference compound SLC-0111.

Chemotherapy is still the standard of care for triple-negative breast cancer (TNBC). Poor prognosis is often ascribed to the emergence of drug resistance to therapeutic agents. In order to evaluate the biological effect of CA inhibitors in TNBC cells, we used MDA-MB 231 and BT-549 cells, which are representative of highly aggressive TNBC subtypes. As shown in Figure 5, Panel A, treatment with compound **15** exerted a potent antiproliferative effect on MDA-MB 231 cells. The effect of **15** was significantly higher as compared with the reference compound SLC-0111, an ureido-substituted benzenesulfonamide small molecule inhibitor of CA IX in Phase 1 trials in patients with advanced solid tumors^44^ (Figure 5, Panel B). Similarly, when tested on BT-549 cells, **15**, significantly impaired cell growth (Figure 5, Panel C) as compared with SLC-0111 (Figure 5, Panel D).

#### Human Primary T Lymphocytes

**Figure 6:**
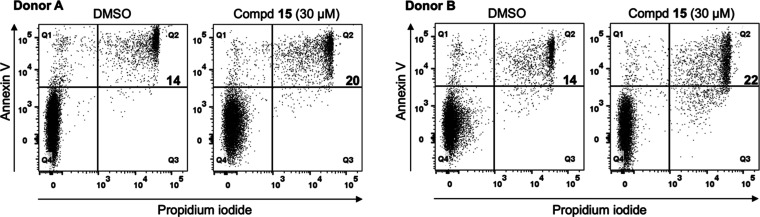
Human primary T cells were treated with **15** at 30 μM or DMSO for 72 h and analyzed for annexin V by flow cytometry. The frequency of annexin V/propidium iodide double positive cells (top right, late phase apoptotic cells) is shown. Data from two different healthy donors (donor A and donor B) are shown.

Potential toxic effects of compound **15** on healthy cells were evaluated by treating human primary T lymphocytes with **15** at 30 μM or with dimethyl sulfoxide (DMSO) as a control vehicle. After 72 h, the frequency of late apoptotic cells was assayed by staining with annexin V and propidium iodide. Flow cytometry analysis showed that compared to the control vehicle, treatment with 30 μM **15** caused very low-level toxicity on healthy/normal cells with only a 6–8% increase in cell death (Figure 6).

#### Detection of Markers of Apoptosis in **15**-Treated HCT116 Cells

**Figure 7:**
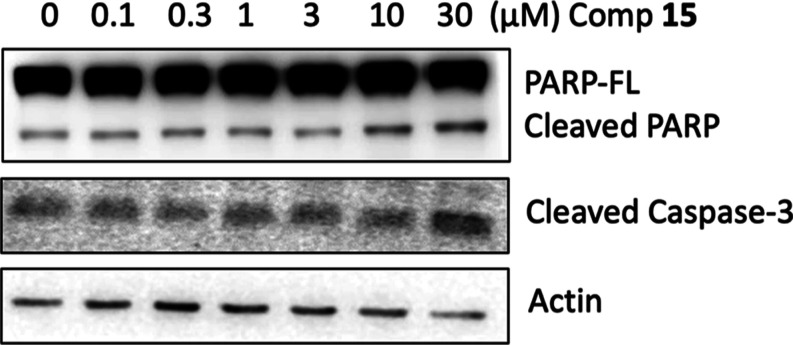
Total and cleaved PARP levels and cleaved caspase-3 levels in HCT116 cells after a 72 h treatment with increasing concentrations of compound **15**.

The inhibitory effect of compound **15** on cell growth could be attributed to the induction of programmed cell death. Detection of cleaved poly(ADPribose) polymerase (PARP) by antibodies is considered a prominent marker of apoptosis. The cleavage of PARP-1 during apoptosis produces two terminal fragments: an 89 kDa C-terminal fragment that contains the catalytic domain and a 24 kDa N-terminal fragment containing the DNA-binding domain. Overexpression of the 24 kDa N-terminal fragment inhibits DNA repair and ADPribose formation and stimulates apoptosis,^45^ with consequent increase of PARP full-length/cleaved ratio and a darker cleaved band. During apoptosis, caspase-3 is a major cause of proteolysis. Detection of cleaved caspase-3 is also a reliable marker of apoptosis since it allows the quantification of cells that are dying or have died by apoptosis.^46^ Treatment of HCT116 cells with increasing concentrations of compound **15** for 72 h highlighted increments of cleaved PARP and cleaved caspase-3 (Figure 7, and Figures S3 and S4, Supporting Information).

#### Inhibition of the Wnt/β-Catenin Signaling Pathway by **15**

**Figure 8:**
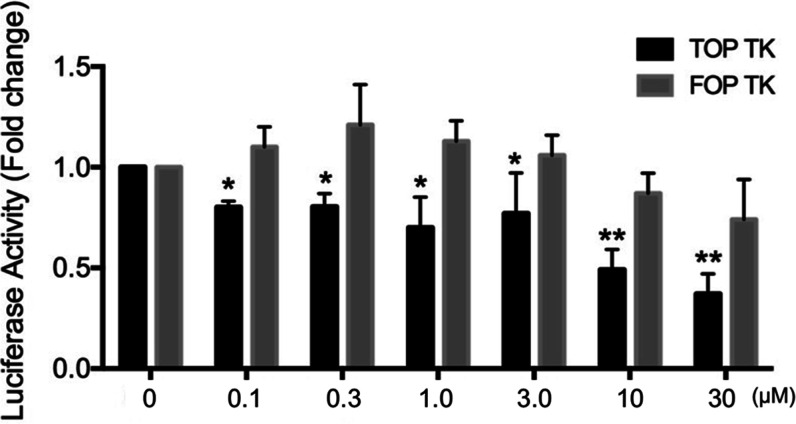
HEK-293 cells were transfected with luciferase-based vectors and treated with LiCl (50 mM) together with increasing concentrations of compound **15**. Cells were harvested 24 h post-treatment and assayed for luciferase activity. Inhibition levels calculated as the luciferase/renilla ratio of the treated samples vs the luciferase/renilla ratio of the untreated (control) samples. Data are represented as the mean ± SD of three independent experiments, each performed in triplicate. *p < 0.05 and **p < 0.01, as determined by analysis of variance (ANOVA).

We wondered whether the observed inhibitory effect of compound **15** on CRC cell viability could be attributed to its ability to affect Wnt signaling. To this end, we first transfected human embryonic kidney (HEK) 293T cells with a reporter vector (M50 Super 8x TOPFlash) containing eight repeats of TCF/LEF-binding sites, or its negative control containing the mutated TCF/LEF binding sites (M51 Super 8x FOPFlash). After transfection, cells were treated with the GSK3 inhibitor LiCl to activate the Wnt pathway and incubated with increasing concentrations of **15**. As shown in Figure 8, the compound caused a significant and dose-dependent inhibition of TOP reporter activity but did not significantly prevent the activity of the FOP reporter, indicating the specificity of the effect.

**Figure 9:**
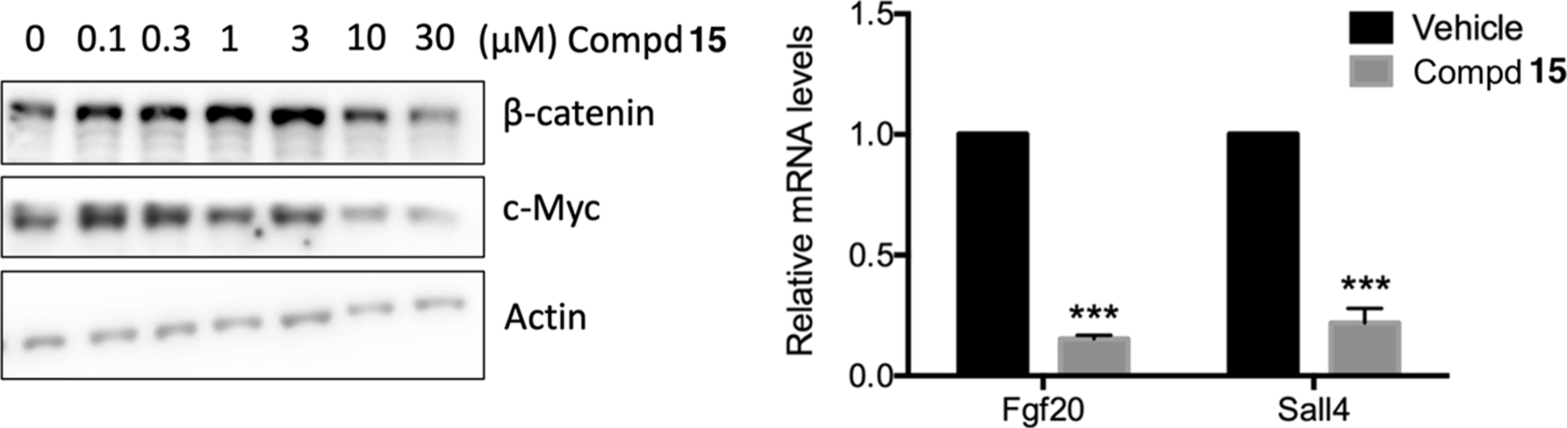
Left panel. HCT116 cells were treated with LiCl (50 mM) and compound **15** at the indicated concentrations for 24 h. β-Catenin and c-Myc levels were analyzed by Western blot. Actin was used as a loading control. Right panel. *Fgf20* or *Sall4* mRNA levels were measured by qPCR and normalized to the expression of β-actin mRNA and expressed as a fold change relative to the control sample. Results represent the mean ± SD of three independent experiments, each performed in triplicate. ***p < 0.001, as determined by the *t*-test.

Treatment of HCT116 CRC cells with increasing concentrations of **15** resulted in a marked reduction of β-catenin and of its target gene MYC (Figure 9, left panel). Furthermore, the compound significantly reduced the expression of two additional Wnt target genes,^47^*Ffg20* and *Sal4* (Figure 9, right panel). Together, these data support the hypothesis that **15** exerts its inhibitory effect on the CRC cell viability and growth by preventing the activation of Wnt/β-catenin signaling.

#### Inhibition of P-gp by **15**

To test the efficacy of compound **15**, we chose the DOX-sensitive colon cancer HT29 cells, a cell line expressing low levels of P-gp, and its resistant counterpart, the cell line HT29/DX, selected stepwise in media with increasing concentrations of DOX resulting in expression of a high level of P-gp.^48^ As a cancer model, the HT29 and HT29/DX pair has been extensively used and characterized for DOX resistance and pharmacological efficacy by our group,^49,50^ and the cell line pair has important translational potential. However, HT29/DX cells also express other ABC transporters including MRP1, MRP2, MRP3, MRP5, and BCRP.^48^ Hence, to better elucidate the effect that the compound under study here has on P-gp, we also included the canine kidney MDCK cell line, devoid of any transporter, and the MDCK/P-gp cell line, overexpressing human P-gp only,^51^ as an internal control. In a preliminary experiment, we measured the cytotoxicity of compound **15** alone. Compound **15** reduced cell viability in the four cell lines, suggesting that there was not a cell-dependent effect or a different behavior between DOX-sensitive and resistant cell lines. The viability was >75% with 1–100 nM **15** (Figure S5, Supporting Information). We worked at 1, 10, and 100 nM concentrations in the subsequent experiments to avoid any bias related to the intrinsic cytotoxicity induced by the compound.

**Figure 10:**
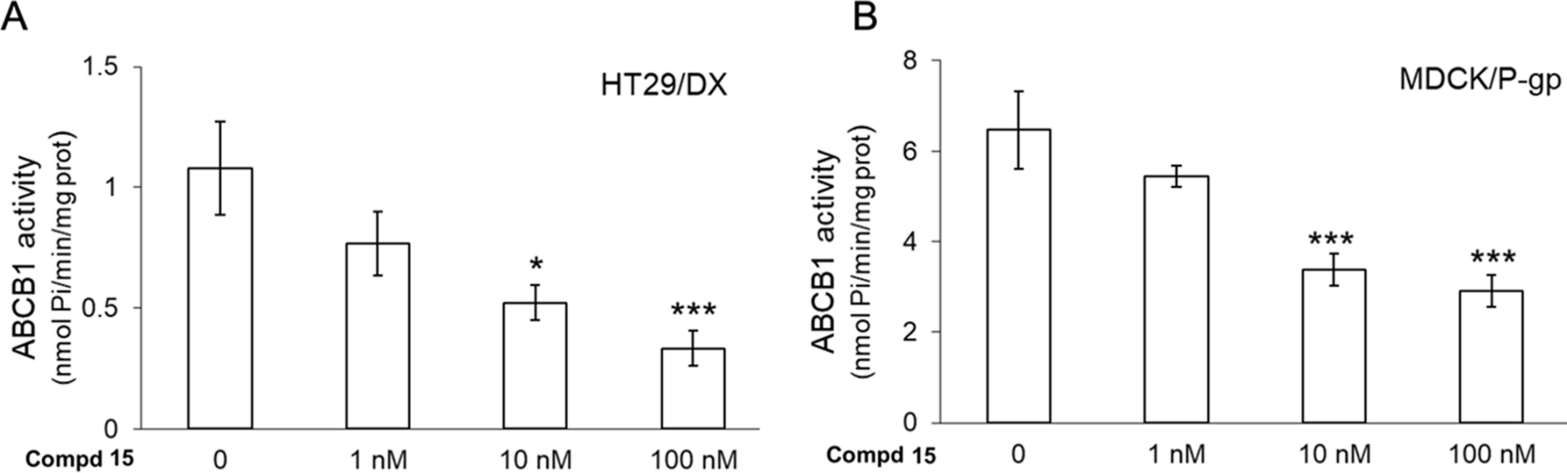
P-gp ATPase activity, measured spectrophotometrically on the protein immune-purified from HT29/DX cells (A) or MDCK/P-gp (B) cells, treated 3 h without (“0”) or with compound **15** at 1, 10, or 100 nM. H. Data are means ± SD (*n* = 3). *p < 0.05 and ***p < 0.001: vs untreated (“0”) cells.

To evaluate the impact as chemosensitizer agents, we first measured the intracellular accumulation of DOX, a typical substrate of P-gp.^52^ As expected, HT29 had a basally higher accumulation of the drug compared to the resistant counterpart HT29/DX cells. Similarly, the intracellular amount of DOX in MDCK cells was significantly higher than that in MDCK/P-gp cells (Figure S6, Supporting Information). Compound **15** had no effects on the amount of DOX retained within HT29 cells, nor in MDCK/P-gp cells, where **15** did not change the intracellular amount of the drug. DOX was basally higher in MDCK/P-gp cells than that in HT29 cells: this difference can be explained because HT29 cells also have other ABC transporters (as MRP1) that, although expressed at low levels, can efflux DOX, while MDCK cells lack other transporters. Differently from what we observed in sensitive cells, **15** induced a strong dose-dependent increase in the DOX accumulation in HT29/DX cells, reaching the same level of intracellular drug detected in sensitive HT29 cells when used at 100 nM. This stronger increase in resistant cells is likely due to their higher levels of the ABC transporters that are the putative targets of compound **15**. Indeed, the higher the level of the transporters, the higher the inhibition achieved by the compounds and greater the increase on DOX retention. Part of the effect of **15** is mediated by the inhibition of P-gp, as indicated by its efficacy in increasing DOX accumulation in MDCK/P-gp cells, where no other transporters than P-gp are present. In this model, also at the highest concentration (100 nM), the compound was not able to fully restore the accumulation of the drug to the intracellular level of MDCK cells, which were devoid of P-gp. This result can be explained at least by following reasons: (1) the higher catalytic efficiency of the protein in MDCK/P-gp and HT29/DX cells (Figure 10) and (2) the possible targeting of other ABC transporters, present in HT29/DX cells but not in MDCK/P-gp cells.

In determining DOX resistance,^52^ we next investigated if **15** was able to inhibit the catalytic cycle of this transporter. We immunopurified the protein from HT29/DX and MDCK/P-gp cell lines, and we measured the rate of ATP hydrolysis, a step necessary to efflux DOX and considered an index of P-gp activity (Table S2, Supporting Information).^53^ As shown in Figure 10, P-gp extracted from MDCK/P-gp cells was more active than the protein extracted from the HT29/DX cells. However, in both cell lines, compound **15** reduced P-gp activity, starting at 10 nM concentration. This result suggests that the compound acts as P-gp inhibitors.

**Figure 11:**
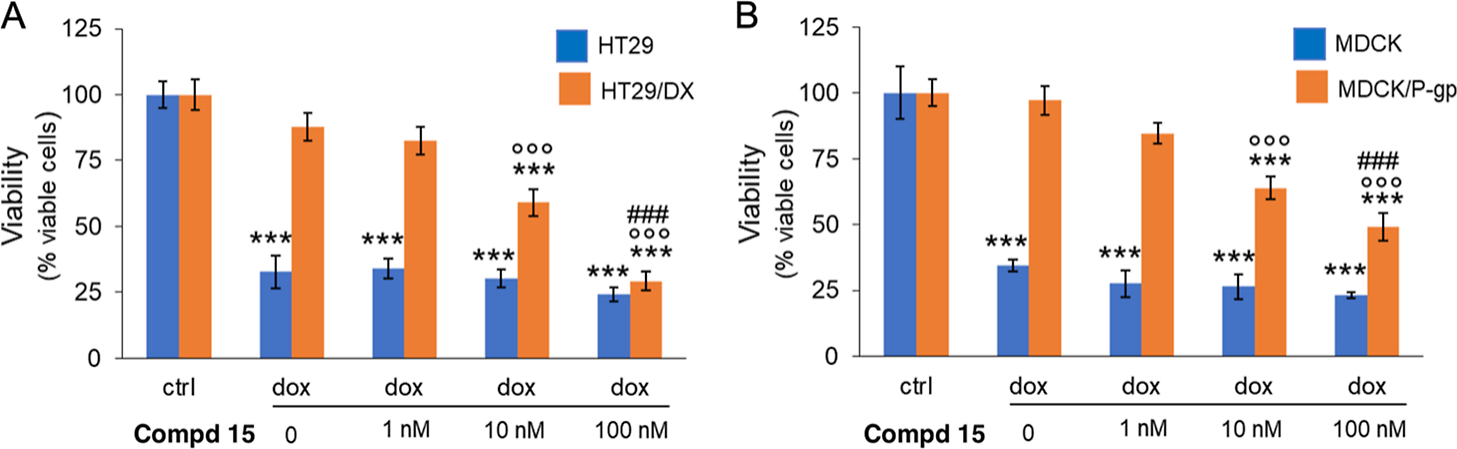
Viability of HT29 and HT29/DX (A), MDCK and MDCK/P-gp (B) cells, incubated with fresh medium (ctrl), 5 μM DOX (dox), alone or coincubated with 1, 10, or 100 nM of **15** for 72 h, measured with a spectrophotometric assay. Data are means ± SD (*n* = 4). ***p < 0.001: versus untreated HT29 or MDCK cells (“ctrl”); °*p* < 0.001: versus untreated HT29/DX or MDCK/P-gp cells (“ctrl”); and ^###^*p* < 0.001: versus dox-treated HT29/DX or MDCK/P-gp cells.

Finally, we tested the potential of compound **15** to reverse resistance to DOX in terms of cytotoxicity, by measuring cell viability in cells coincubated with both the compound and 5 μM DOX, a concentration already used to discriminate sensitive and resistant cells.^54^ Also, in our experimental setting, DOX reduced the viability of sensitive HT29 and MDCK cells below 30%, but it did not affect the viability in resistant HT29/DX and MDCK/P-gp cells (Figure 11). In HT29 and MDCK cells, **15** did not further reduce cell viability compared with DOX alone, consistent with the absence of a significant increase in intracellular retention of the drug. By contrast, in HT29/DX and MDCK/P-gp cells, compound **15** decreased cell viability in a dose-dependent manner, restoring the sensitivity to DOX. Particularly, in the HT29/DX model, the combination of DOX plus **15** at 100 nM produced the same decrease in cell viability achieved by DOX alone in the respective chemosensitive cells, confirming that this setting fully overcame the resistance to DOX.

#### Drug-like Properties of Compound **15**

**Figure 12:**
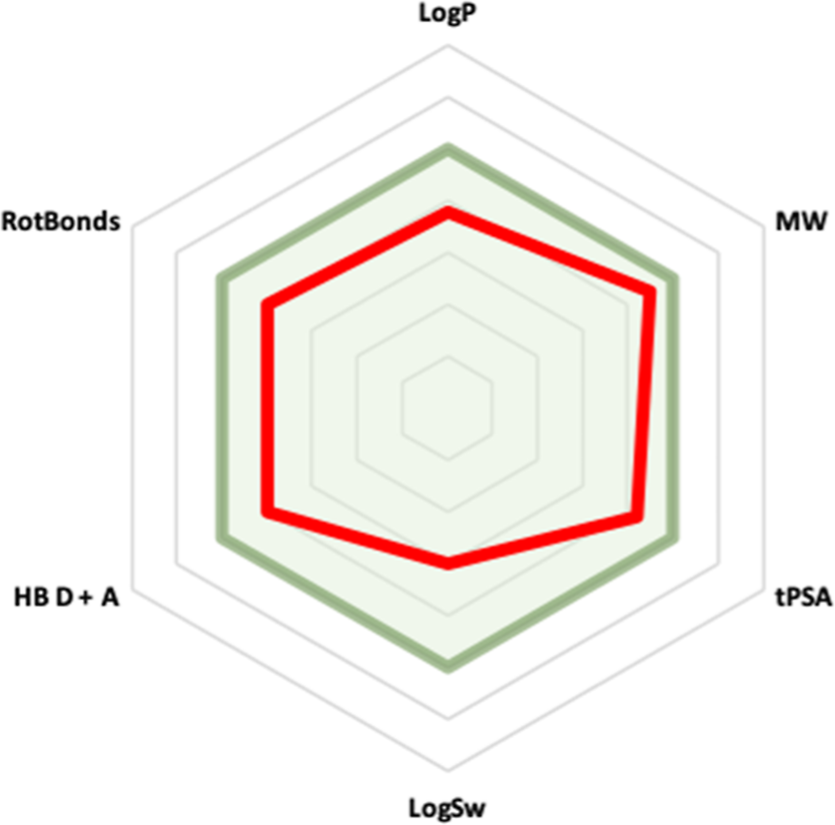
Radar plot of drug-like properties of compound **15**. The light green colored zone represents the suitable physicochemical space for oral bioavailability. −1 < Log *P* < 5; 150 < MW < 500; 20 Å^2^ < tPSA < 140 Å^2^; −10 < Log Sw < 0; 0 < HB D + A < 10; and 0 < RotBonds < 10. The red line represents values for derivative **15**.

Representative drug-like properties of compound **15** were calculated on the SwissADME website. There were no violations of the Lipinski^55^ and Veber^56^ rules; compound **15** was predicted to have good bioavailability after oral administration (Table 4 and Figure 12).

**Table 4:** Drug-like Properties of Compound **15**

comp	Log *P*^a^	MW^b^	Log Sw^c^	tPSA^d^	HBA^e^	HBD^f^	rot^g^	Lipinski^h^	Veber^i^
**15**	3.77	492.54	–5.23	118.23	7	1	8	0	0

a Logarithm of the partition coefficient between *n*-octanol and water computed by the XLOGP3 method.

b Molecular weight.

c LogSw represents the logarithm of compound water solubility computed by the ESOL method. Log Sw values predicted compounds likely to be >−10: insoluble, >−6: poorly soluble, > −4: moderately soluble, >−2: soluble, and >0: high soluble.

d Molecular polar surface area, this parameter has been shown to correlate with human intestinal absorption (<140 Å^2^).

e Number H-bond acceptors.

f Number H-bond donors.

g Number of rotatable bonds.

h Violation of the Lipinski rule of five (MW < 500; log *P* < 5; HBD ≤ 10; HBA ≤ 5).

i Veber’s rule matching (Rot <10 and tPSA < 140 Å^2^).

## Conclusions

We synthesized new pyrrole and indole CA inhibitors **10**–**21** by modulating the scaffold of previously reported tubulin polymerization inhibitors **2–4**.^21,22^ Derivatives bearing the sulfonamide at the *para* position of the *N*1-phenyl ring showed strong inhibition of the hCA isoforms I, II, IX, and XII with *K*_i_ values at nanomolar concentrations. Compounds **11**, **15**, and **19** characterized by the presence of both sulfonamidophenyl and 3,4,5-trimethoxyphenyl substituents at positions 1 and 3, respectively, of the heterocycle were the strongest hCA inhibitors within the series. In particular, **11** inhibited the hCA II and hCA IX isoforms with *K*_i_ values of 10.2 and 11.5 nM, respectively; **15** inhibited the hCA XII with *K*_i_ of 6.8 nM; and **19** inhibited the hCA IX with *K*_i_ of 7.3 nM. Conversely, **11**, **15**, and **19** had limited ability to inhibit tubulin polymerization: at 20 μM, **15** and **19** produced only partial inhibition of tubulin assembly, while CSA4 inhibited assembly by over 50% at less than 1 μM. The docking poses of compounds **11**, **15**, and **19** in the binding sites of the four hCA isoforms were consistent with the observed *K*_i_ values. On the other hand, the docking poses of **11**, **15**, and **19** into the colchicine site of tubulin revealed that the sulfonamide group did not form stabilizing contacts, suggesting that the desolvation energy cost was not balanced by the binding energy.

As inhibitors of the HCT116 cells, compounds **15** and **19** were worthy of comparison with 5-FU, and they were remarkably more potent than 5-FU as inhibitors of the SW480 and SW620 cell lines. Compounds **11**, **15**, and **19** inhibited the MCF-7 and OVCAR-8 cell lines with IC_50_ values in the single digit micromolar range and, notably, were almost equipotent against the NCI/ADR-RES DOX-resistant cell line. Compound **15** potently inhibited MDA-MB 231 and BT-549 TNBC cells and was superior to the reference compound SLC-0111.

Compound **15** caused significant suppression of the Wnt-β-catenin signaling pathway, as indicated by inhibition of TOP reporter activity, and its target gene MYC, and significantly reduced the expression of the Wnt target genes *Fgf20* and *Sal4*. Compound **15** induced apoptotic programmed cell death, as demonstrated by the increase of apoptotic markers cleaved PARP and cleaved Caspase-3. Compound **15** restored the sensitivity to DOX in HT29/DX and MDCK/P-gp cells. In the HT29/DX model, **15** at 100 nM in combination with DOX decreased the cell viability achieved by DOX alone in the respective chemosensitive cells.

In summary, we describe the synthesis of new pyrrole and indole derivatives as hCA inhibitors with *K*_i_ values in the nanomolar range. Compound **15**, the most potent hCA XII inhibitor with *K*_i_ = 6.8 nM, caused significant suppression of the Wnt/β-catenin signaling pathway and induced apoptotic cell death in HCT116 cells. Compound **15** restored the sensitivity to DOX in HT29/DX and MDCK/P-gp cells. As a cell growth inhibitor, **15** was equipotent to 5-FU in HCT116 cells and remarkably more potent in the SW480 and SW620 cell lines and inhibited the NCI/ADR-RES DOX-resistant cell line. Against the MDA-MB 231 and BT-549 TNBC cells, **15** was superior to reference compound SLC-0111. Together, these results highlight compound **15** as a novel hCA/β-catenin pathway dual-targeting broad spectrum anticancer agent with a specific potential to restore sensitivity to P-gp expressing cell lines. These findings highlight **15** as a lead compound of a novel class of dual-targeting broad spectrum anticancer agents and will prompt additional studies to explore its potential for the treatment of cancer.

## Experimental Section

### Chemistry

All reagents and solvents were handled according to the material safety data sheet of the supplier and used as purchased without further purification. Organic solutions were dried over anhydrous sodium sulfate. Evaporation of solvents was carried out on a Büchi Rotavapor R-210 equipped with a Büchi V-850 vacuum controller and a Büchi V-700 vacuum pump. Column chromatography was performed on columns packed with silica gel from the Macherey–Nagel (70–230 mesh). Silica gel thin-layer chromatography (TLC) cards from Macherey-Nagel (silica gel-precoated aluminum cards with a fluorescent indicator visualizable at 254 nm) were used for TLC. Developed plates were visualized with a Spectroline ENF 260C/FE UV apparatus. Melting points (mp) were determined on a Stuart Scientific SMP1 apparatus and are uncorrected. Infrared (IR) spectra were recorded on a PerkinElmer Spectrum 100 FT-IR spectrophotometer equipped with a universal attenuated total reflectance accessory, and IR data were acquired and processed by PerkinElmer Spectrum 10.03.00.0069 software. Band position and absorption ranges are given in cm^–1^. Proton nuclear magnetic resonance (^1^H NMR) spectra were recorded with a Bruker Avance (400 MHz) spectrometer in the indicated solvent, and the corresponding fid files were processed with MestreLab Research SL MestreReNova 6.2.1–769 software. Carbon-13 nuclear magnetic resonance (^13^C NMR) spectra were recorded with a Bruker AVANCE (100 MHz) spectrometer in the indicated solvent, and the corresponding FID files were processed by MestreLab Research SL MestreReNova 6.2.1–769 software. Chemical shifts of ^1^H and ^13^C NMR are expressed in δ units (ppm) from tetramethylsilane.

Compound purity was checked by high-pressure liquid chromatography (HPLC). Purity of tested compounds was found to be >95%. The HPLC system used (Thermo Fisher Scientific Inc. Dionex UltiMate 3000) consisted of an SR-3000 solvent rack, an LPG-3400SD quaternary analytical pump, a TCC-3000SD column compartment, a DAD-3000 diode array detector, and an analytical manual injection valve with a 20 μL loop. Samples were dissolved in acetonitrile (1 mg/mL). HPLC analysis was performed by using a Thermo Fisher Scientific Inc. Acclaim 120 C18 column (5 μm, 4.6 mm × 250 mm), at 25 ± 1 °C with an appropriate solvent gradient (acetonitrile/water), flow rate of 1.0 mL/min, and signal detector at 206, 230, 254, and 365 nm were used. Chromatographic data were acquired and processed by Thermo Fisher Scientific Inc. Chromeleon 6.80 SR15 Build 4656 software. UHPLC analysis was carried out on an Accela System Thermo Fisher Scientific (San Jose, CA) which consisted of an Accela 1250 Pump, an Accela autosampler, and an Accela PDA photodiode array detector. Chromatographic data were collected and processed using Thermo Xcalibur Chromatography Manager software, version 1.0. A guard cartridge system (SecurityGuard Ultra UHPLC) has been connected to an analytical column Kinetex 2.6 μm EVO C18 100 Å 100 × 3.0 mm (L. x I.D.), both from Phenomenex, Torrance, CA, USA. All analyses were performed at 30 °C, and the mobile phase was filtered through 0.2 μm Omnipore filters (Merck Millipore, Darmstadt, Germany). The mobile phase was delivered at a total flow rate of 0.6 mL/min. The analyses were carried out in the elution gradient. Specific mobile phase and gradient are reported for each compound in captions. Each analysis was performed in triplicate (Figures S7–S9 and Table S3, Supporting Information). Materials: Acetonitrile (HPLC gradient grade), methanol (HPLC gradient grade), water (HPLC gradient grade), and trifluoroacetic acid (HPLC grade) were purchased from Sigma-Aldrich (St. Louis, MO).

#### General Procedure for the Preparation of Compounds **10**–**21**

##### Example: 4-(3-Benzoyl-1*H*-pyrrol-1-yl)benzenesulfonamide (**10**)

Compound **27** (191 mg, 0,325 mmol) was dissolved in anhydrous THF (5 mL). TBAF solution 1.0 M in THF (1.12 mL) was added dropwise, and then, the mixture was heated under reflux or 4 h. After being cooled, the mixture was diluted with water and extracted with ethyl acetate. The organic layer was washed with brine, dried on anhydrous sodium sulfate, and filtered. Evaporation of the solvent gave a residue that was purified by silica gel column chromatography (Hex/AcOEt, 1:1) to give **10** (74 mg, 70%), mp 204–206 °C (from ethanol). ^1^H NMR (DMSO-*d*_6_, 400 MHz): δ 6.78 (s, 1H), 7.46 (s, 2H), 7.50–7.55 (m, 3H), 7.59–7.62 (m, 1H), 7.80–7.84 (t, *J* = 8 Hz, 4H), 7.90–7.94 (t, *J* = 8 Hz, 3H) ppm. ^13^C NMR (DMSO-*d*_6_, 100 MHz): δ 113.11, 121.19, 122.37, 126.44, 126.59, 128.19, 129.34, 132.83, 139.33, 141.89, 142.24, 190.62 ppm. IR: v 1162, 1640, 2981 cm^–1^.

##### 4-(3-(3,4,5-trimethoxybenzoyl)-1*H*-pyrrol-1-yl)benzenesulfonamide (**11**)

It was synthesized as **10** starting from **28**. Yield 88%, mp 171–174 °C (from ethanol). ^1^H NMR (DMSO-*d*_6_): δ 3.80 (d, *J* = 9.2 Hz, 6H), 3.87 (s, 3H), 7.10 (br s, disappeared after treatment with D_2_O, 2H), 7.19–7.24 (m, 2H), 7.25–7.35 (m, 3H), 7.60 (d, *J* = 8.5 Hz, 2H), 7.62 (d, *J* = 2.4 Hz, 1H), 8.04 ppm (d, *J* = 8.5 Hz, 2H). ^13^C NMR (DMSO-*d*_6_, 100 MHz): δ 56.45, 60.56, 106.72, 112.87, 120.80, 121.96, 126.20, 126.30, 127.79, 134.63, 141.15, 141.63, 142.28, 153.14, 188.70 ppm. IR ν 1463 and 2922 cm^–1^.

##### 3-(3-(3,4,5-Trimethoxybenzoyl)-1*H*-pyrrol-1-yl)benzenesulfonamide (**12**)

It was synthesized as **10** starting from **29**. Yield 90%, mp 169–171 °C (from ethanol). ^1^H NMR (DMSO-*d*_6_, 400 MHz): δ 3.77 (s, 3H), 3.86 (s, 6H), 6.86 (s, 1H), 7.15 (s, 2H), 7.45 (s, 2H), 7.58–7.59 (m, 1H), 7.69–7.73 (t, *J* = 8 Hz, 1H), 7.77–7.79 (d, *J* = 8 Hz, 1H), 7.98–8.00 (d, *J* = 8 Hz, 1H), 8.08–8.10 (d, *J* = 8 Hz, 2H) ppm. ^13^C NMR (DMSO-*d*_6_, 100 MHz): δ 56.00, 66.11, 106.31, 112.36, 117.41, 121.49, 123.38, 123.61, 125.70, 125.74, 130.68, 134.21, 139.24, 140.75, 145.61, 152.68, 188.21 ppm. IR: ν 1641 and 2982 cm^–1^.

##### 2-(3-(3,4,5-Trimethoxybenzoyl)-1*H*-pyrrol-1-yl)benzenesulfonamide (**13**)

It was synthesized as **10** starting from **30**. Yield 88%, mp 64–66 °C (from ethanol). ^1^H NMR (DMSO-*d*_6_, 400 MHz): δ 3.73 (s, 3H), 3.85 (s, 6H), 6.73–6.74 (m, 1H), 7.10–7.11 (m, 1H), 7.15 (s, 2H), 7.51–7.53 (d, *J* = 8 Hz, 1H), 7.61–7.75 (m, 5H), 8.06–8.08 (d, *J* = 8 Hz, 1H) ppm. ^13^C NMR (DMSO-*d*_6_, 100 MHz): δ 55.99, 60.09, 106.21, 110.15, 123.94, 125.60, 128.20, 128.95, 129.64, 131.08, 134.69, 136.63, 139.45, 140.53, 152.63, 189.30 ppm. IR: ν 1644 and 2985 cm^–1^.

##### 4-(3-Benzoyl-4-phenyl-1*H*-pyrrol-1-yl)benzenesulfonamide (**14**)

It was synthesized as **10** starting from **33**. Yield 98%, mp 211–214 °C (from ethanol). ^1^H NMR (DMSO-*d*_6_, 400 MHz): δ 7.21–7.24 (t, *J* = 8 Hz, 1H), 7.28–7.32 (t, *J* = 8 Hz, 2H), 7.41–7.44 (d, *J* = 12 Hz, 4H), 7.47–7.51 (t, *J* = 8 Hz, 2H), 7.59–7.63 (t, *J* = 8 Hz, 1H), 7.86.7.87 (m, 3H), 7.91–7.94 (m, 3H), 7.98–8.00 (d, *J* = 8 Hz, 2H) ppm. ^13^C NMR (DMSO-*d*_6_, 100 MHz): δ 120.14, 120.25, 123.38, 126.39, 126.78, 127.33, 127.95, 128.01, 128.28, 128.41, 129.35, 132.29, 133.95, 138.84, 140.94, 141.77, 190.25 ppm. IR: ν 1631 and 3218 cm^–1^.

##### 4-(3-Phenyl-4-(3,4,5-trimethoxybenzoyl)-1*H*-pyrrol-1-yl)benzenesulfonamide (**15**)

It was synthesized as **10** starting from **34**. Yield 80%, mp 223–225 °C (from ethanol). ^1^H NMR (DMSO-*d*_6_): δ 3.78 (s, 3H), 3.84 (s, 6H), 7.19 (br s, disappeared after treatment with D_2_O, 2H), 7.25–7.26 (m, 1H), 7.29–7.37 (m, 2H), 7.49 (m, 4H), 7.93–7.99 (m, 3H), 8.06–8.09 (m, 2H), 8.13 ppm (d, *J* = 2.4 Hz, 1H). ^13^C NMR (DMSO-*d*_6_, 100 MHz): δ 55.91, 60.08, 106.98, 119.99, 120.07, 123.44, 126.35, 126.39, 127.31, 127.95, 127.98, 128.24, 133.74, 134.03, 140.93, 141.14, 141.65, 152.48, 189.04 ppm. IR ν 1463 and 2922 cm^–1^.

##### 3-(3-Phenyl-4-(3,4,5-trimethoxybenzoyl)-1*H*-pyrrol-1-yl)benzenesulfonamide (**16**)

It was synthesized as **10** starting from **35**. Yield 71%, mp 114–116 °C (from ethanol). ^1^H NMR (DMSO-*d*_6_, 400 MHz): δ 3.73 (s, 3H), 3.78 (s, 6H), 7.14 (s, 2H), 7.20–7.24 (t, *J* = 8 Hz, 1H), 7.28–7.32 (t, *J* = 8 Hz, 2H), 7.40–7.42 (d, *J* = 8 Hz, 2H), 7.45 (s, 2H), 7.70–7.74 (t, *J* = 8 Hz,1H), 7.78–7.80 (d, *J* = 8 Hz, 1H), 7.81 (s, 1H), 8.01 (s, 1H), 8.04–8.06 (d, *J* = 8 Hz, 1H), 8.18 (s, 1H) ppm. ^13^C NMR (DMSO-*d*_6_, 100 MHz): δ 55.92, 60.09, 107.03, 117.10, 120.04, 123.24, 126.37, 127.94, 128.97, 130.64, 133.83, 134.04, 139.02, 141.18, 145.64, 152.49, 189.05 ppm. IR: ν 1164 and 3220 cm^–1^.

##### 2-(3-Phenyl-4-(3,4,5-trimethoxybenzoyl)-1*H*-pyrrol-1-yl)benzenesulfonamide (**17**)

It was synthesized as **10** starting from **36**. Yield 80% as an oil. ^1^H NMR (DMSO-*d*_6_, 400 MHz): δ 3.73 (s, 3H), 3.83 (s, 6H), 7.20–7.23 (m, 3H), 7.28–7.32 (m, 3H), 7.42–7.44 (d, *J* = 8 Hz, 2H), 7.52–7.53 (d, *J* = 4 Hz, 1H), 7.62–7.64 (d, *J* = 8 Hz, 1H), 7.67–7.71 (t, *J* = 8 Hz, 3H), 7.74–7.77 (t, *J* = 8 Hz, 1H), 8.07–8.09 (d, *J* = 8 Hz, 1H) ppm. ^13^C NMR (DMSO-*d*_6_, 100 MHz): δ 55.99, 60.09, 107.21, 110.25, 119.12, 120.33, 122.94, 125.80, 126.78, 128.40, 128.95, 130.64, 131.08, 134.96, 136.53, 139.51, 141.53, 150.63, 190.30 ppm. IR: ν 1635 and 3222 cm^–1^.

##### 4-(3-Benzoyl-1H-indol-1-yl)benzenesulfonamide (**18**)

It was synthesized as **10** starting from **39**. Yield 82%, mp 249–252 °C (form ethanol). ^1^H NMR (DMSO-*d*_6_, 400 MHz): δ 7.39–7.41 (m, 2H), 7.54–7.58 (m, 4H), 7.62–7.67 (m, 2H), 7.91–7.96 (t, *J* = 12 Hz, 3H), 8.03–8.05 (d, *J* = 8 Hz, 2H), 8.24 (s, 1H), 8.38–8.40 (m, 1H) ppm. ^13^C NMR (DMSO-*d*_6_, 100 MHz): δ 111.20, 116.45, 122.23, 123.39, 124.59, 125.18, 127.46, 128.66, 128.83, 131.75, 136.05, 137.30, 139.76, 140.32, 143.09, 190.06 ppm. IR: ν 1630 and 3294 cm^–1^.

##### 4-(3-(3,4,5-Trimethoxybenzoyl)-1*H*-indol-1-yl)benzenesulfonamide (**19**)

It was synthesized as **10** starting from **40**. Yield 80%, mp 127–130 °C (from ethanol). ^1^H NMR (DMSO-*d*_6_, 400 MHz): δ 3.77 (s, 3H), 3.87 (s, 6H), 7.21 (br s, disappeared after treatment with D_2_O, 2H), 7.38–7.43 (m, 2H), 7.55 (s, 2H), 7.66–7.71 (m, 1H), 7.93–7.95 (m, 2H), 8.02–8.06 (m, 2H), 8.36–8.40 (m, 1H) and 8.42 (s, 1H) ppm. ^13^C NMR (DMSO-*d*_6_, 100 MHz): δ 56.54, 60.56, 106.80, 111.59, 116.88, 122.60, 123.68, 124.90, 125.62, 127.82, 128.00, 135.50, 136.50, 137.51, 140.73, 140.97, 143.41, 153.19, 189.50 ppm. IR ν 1463 and 2922 cm^–1^.

##### 3-(3-(3,4,5-Trimethoxybenzoyl)-1*H*-indol-1-yl)benzenesulfonamide (**20**)

It was synthesized as **10** starting from **41**. Yield 71%, mp 224–226 °C (from ethanol). ^1^H NMR (DMSO-*d*_6_): δ 3.77 (s, 3H), 3.86 (s, 6H), 7.21 (s, 2H), 7.39–7.42 (m, 2H), 7.53 (s, 2H), 7.62–7.64 (m, 1H), 7.81–7.85 (t, *J* = 8.0 Hz, 1H), 7.92–7.97 (m, 2H), 8.09 (s, 1H), 8.36–8.38 (m, 2H) ppm. ^13^C NMR (DMSO-*d*_6_, 100 MHz): δ 56.12, 60.13, 106.41, 110.87, 116.35, 121.72, 123.23, 124.71, 127.43, 128.25, 130.95, 135.09, 136.15, 137.12, 138.02, 140.57, 145.47, 152.75, 190.06 ppm. IR: ν 1632 and 3298 cm^–1^.

##### 2-(3-(3,4,5-Trimethoxybenzoyl)-1*H*-indol-1-yl)benzenesulfonamide (**21**)

It was synthesized as **10** starting from **42**. Yield 79%, mp 239–241 °C (from ethanol). ^1^H NMR (DMSO-*d*_6_, 400 MHz): δ 3.74 (s, 3H), 3.86 (s, 6H), 7.00–7.02 (d, *J* = 8 Hz, 1H), 7.20 (s, 2H), 7.25–7.29 (t, *J* = 8 Hz, 1H), 7.31–7.35 (t, *J* = 8 Hz, 1H), 7.58.7.60 (m, 1H), 7.65 (s, 2H), 7.78–7.83 (m, 2H), 8.09 (s, 1H), 8.15–8.18 (m, 1H), 8.32–8.34 (d, *J* = 8 Hz, 1H) ppm. ^13^C NMR (DMSO-*d*_6_, 100 MHz): δ 56.02, 60.10, 106.21, 111.24, 115.15, 121.66, 122.53, 123.81, 126.49, 128.50, 129.81, 130.96, 133.22, 134.27, 135.36, 138.80, 140.11, 140.36, 141.20, 152.70, 189.09 ppm. IR: ν 1167 and 3263 cm^–1^.

#### General Procedure for the Preparation of Compounds **27**–**30**, **33**–**36**, and **39**–**42**

##### Example: 4-(3-benzoyl-1H-pyrrol-1-yl)-*N*,*N*-bis((2-(trimethylsilyl)ethoxy)methyl)benzenesulfonamide (**27**)

An overdried tube was charged at room temperature with **22** (82 mg, 0.479 mmol), **24** (285.45 mg, 0.575 mmol), CuI (45.71 mg, 0.24 mmol), Cs_2_CO_3_ (234 mg, 0.718 mmol), and 1,10-phenanthroline (8.64 mg, 0.048) in dioxane (6 mL). The mixture was placed into the microwave cavity (close vessel mode, PMAX 200 PSI). Microwave irradiation of 50 W was used at first, then rose up to 200 W. The temperature was ramped from 25 to 210 °C. Once it was reached, taking around 7 min, the mixture was held at this temperature for 40 min. After cooling, the mixture was diluted with water and extracted with ethyl acetate. The organic layer was washed with brine, dried on anhydrous sodium sulfate and filtered. Removal of the solvent gave a residue that was purified by silica gel column chromatography (Cyhex/AcOEt, 8:2) to give **27** (140 mg, 50%) as an oil. ^1^H NMR (CDCl_3_, 400 MHz): δ 0.04 (s, 18H), 0.82–0.86 (t, *J* = 8 Hz, 4H), 3.47–3.51 (t, *J* = 8 Hz, 4H), 4.78 (s, 4H), 6.90–6.91 (m, 1H), 7.15–7.16 (m, 1H), 7.46–7.56 (m, 5H), 7.64 (s, 1H), 7.86–7.88 (d, *J* = 8 Hz, 2H), 7.99–8.01 (d, *J* = 8 Hz, 2H) ppm. IR ν 1464 cm^–1^.

##### 4-(3-(3,4,5-Trimethoxybenzoyl)-1*H*-pyrrol-1-yl)-*N*,*N*-bis((2 (trimethylsilyl)ethoxy)methyl)benzenesulfonamide (**28**)

It was synthesized as **27** starting from **23** and **24**. Yield 58% as an oil. ^1^H NMR (DMSO-*d*_6_, 400 MHz): δ 0.00 (s, 18H), 0.81–0.86 (m, 4H), 3.46 (m, 4H), 3.85 (s, 3H), 3.94 (s, 6H), 4.84 (s, 4H), 6.94 (m, 1H), 7.21 (s, 2H), 7.76–7.78 (m, 1H), 8.02–8.05 (m, 2H), 8.08–8.10 (m, 2H) and 8.25–8.26 ppm (m, 1H). IR ν 1463 and 2922 cm^–1^.

##### 3-(3-(3,4,5-Trimethoxybenzoyl)-1*H*-pyrrol-1-yl)-*N*,*N*-bis((2 (trimethylsilyl)ethoxy)methyl)benzenesulfonamide (**29**)

It was synthesized as **27** starting from **23** and **25**. Yield 58% as an oil. ^1^H NMR (DMSO-*d*_6_, 400 MHz): δ 0.11 (s, 18H), 0.68–0.72 (t, *J* = 8 Hz, 4H), 3.35–3.40 (t, *J* = 8 Hz, 4H), 3.77 (s, 3H), 3.86 (s, 6H), 4.78 (s, 4H), 6.83 (s, 1H), 7.15 (s, 2H), 7.63 (s, 1H), 7.69–7.73 (t, *J* = 8 Hz, 1H), 7.81–7.83 (d, *J* = 8 Hz, 1H), 8.05–8.07 (d, *J* = 8 Hz, 1H), 8.11 (s, 1H), 8.15 (s, 1H) ppm. IR ν 1464 and 2919 cm^–1^.

##### 2-(3-(3,4,5-Trimethoxybenzoyl)-1*H*-pyrrol-1-yl)-*N*,*N*-bis((2-(trimethylsilyl)ethoxy)methyl)benzenesulfonamide (**30**)

It was synthesized as **27** starting from **23** and **26**. Yield 24% as an oil. ^1^H NMR (DMSO-*d*_6_, 400 MHz): δ 0.09 (s, 18H), 0.65–0.69 (t, *J* = 8 Hz, 4H), 3.28–3.32 (t, *J* = 8 Hz, 4H), 3.71 (s, 3H), 3.84 (s, 6H), 4.40 (s, 4H), 6.78 (s, 1H), 7.00–7.02 (t, *J* = 4 Hz, 1H), 7.15 (s, 2H), 7.55–7.57 (d, *J* = 8 Hz, 1H), 7.60 (s, 1H), 7.70–7.74 (t, *J* = 8 Hz, 1H), 7.80–7.83 (t, *J* = 4 Hz, 1H), 8.10–8.12 (d, *J* = 8 Hz, 1H) ppm. IR ν 1464 and 2919 cm^–1^.

##### 4-(3-Benzoyl-4-phenyl-1*H*-pyrrol-1-yl)-*N*,*N*-bis((2-(trimethylsilyl)ethoxy)methyl)benzenesulfonamide (**33**)

It was synthesized as **27** starting from **31** and **24**. Yield 33% as an oil. ^1^H NMR (DMSO-*d*_6_, 400 MHz): δ 0.08 (s, 18H), 0.73–0.78 (t, *J* = 12 Hz, 4H), 3.35–3.39 (t, *J* = 8 Hz, 4H), 4.75 (s, 4H), 7.20–7.24 (t, *J* = 8 Hz, 1H), 7.28–7.32 (t, *J* = 8 Hz, 2H), 7.40–7–42 (d, *J* = 8 Hz, 2H), 7.46–7.50 (t, *J* = 8 Hz, 2H), 7.59–7.63 (t, *J* = 8 Hz, 1H), 7.83–7.85 (d, *J* = 8 Hz, 2H), 7.88–7.89 (m, 1H), 7.94–7.96 (d, *J* = 8 Hz, 3H), 8.01–8.03 (d, *J* = 8 Hz, 2H), ppm. IR: ν 1461 and 2923 cm^–1^.

##### 4-(3-Phenyl-4-(3,4,5-trimethoxybenzoyl)-1*H*-pyrrol-1-yl)-*N*,*N*-bis((2-(trimethylsilyl)ethoxy)methyl)benzenesulfonamide. (**34**)

It was synthesized as **27** starting from **32** and **24**. Yield 65%, oil. ^1^H NMR (DMSO-*d*_6_, 400 MHz): δ 0.03 (s, 18H), 0.84–0.88 (m, 4H), 3.50–3.54 (m, 4H), 3.78 (s, 6H), 3.86 (s, 3H), 4.79 (s, 4H), 7.10 (s, 2H), 7.18–7.26 (m, 2H), 7.26–7.28 (m, 2H), 7.32–7.35 (m, 2H), 7.56–7.58 (m, 2H), 7.63 (d, *J* = 2.5 Hz, 1H), 8.02–8.05 ppm (m, 2H). IR ν 1463 and 2922 cm^–1^.

##### 3-(3-Phenyl-4-(3,4,5-trimethoxybenzoyl)-1*H*-pyrrol-1-yl)-*N*,*N*-bis((2-(trimethylsilyl)ethoxy)methyl)benzenesulfonamide (**35**)

It was synthesized as **27** starting from **32** and **25**. Yield 61% as an oil. ^1^H NMR (DMSO-*d*_6_, 400 MHz): δ 0.11 (s, 18H), 0.69–0.73 (t, *J* = 8 Hz, 4H), 3.36–3.40 (t, *J* = 8 Hz, 4H), 3.73 (s, 3H), 3.77 (s, 6H), 3.79 (s, 4H), 7.13 (s, 2H), 7.19–7.23 (t, *J* = 8 Hz, 1H), 7.27–7.31 (t, *J* = 8 Hz, 2H), 7.38–7.39 (d, *J* = 4 Hz, 2H), 7.69–7.73 (t, *J* = 8 Hz, 1H), 7.80–7.82 (d, *J* = 8 Hz, 1H), 7.86–7.87 (d, *J* = 4 Hz, 1H), 8.08 (m, 1H), 8.11–8.13 (d, *J* = 8 Hz, 1H), 8.18 (s, 1H) ppm. IR ν 1463 and 2922 cm^–1^.

##### 2-(3-Phenyl-4-(3,4,5-trimethoxybenzoyl)-1*H*-pyrrol-1-yl)-*N*,*N*-bis((2-(trimethylsilyl)ethoxy)methyl)benzenesulfonamide (**36**)

It was synthesized as **27** starting from **32** and **26**. Yield 24% as an oil. ^1^H NMR (DMSO-*d*_6_, 400 MHz): δ 0.10 (s, 8H), 0.64–0.68 (t, *J* = 8 Hz, 4H), 3.29–3.33 (t, *J* = 8 Hz, 4H), 3.72 (s, 3H), 3.80 (s, 6H), 4.46 (s, 4H), 7.18–7.19 (m, 3H), 7.21–7.24 (t, *J* = 4 Hz, 1H), 7.28–7.32 (t, *J* = 8 Hz, 2H), 7.37–7.39 (d, *J* = 8 Hz, 2H), 7.48–7.49 (d, *J* = 4 Hz, 1H), 7.67–7.69 (d, *J* = 8 Hz, 1H), 7.71–7.75 (t, *J* = 8 Hz, 1H), 7.82–7.86 (t, *J* = 8 Hz, 1H), 8.12–8.14 (d, *J* = 8 Hz, 1H) ppm. IR ν 1464 and 2919 cm^–1^.

##### 4-(3-Benzoyl-1*H*-indol-1-yl)-*N*,*N*-bis((2 (trimethylsilyl)ethoxy)methyl)benzenesulfonamide (**39**)

It was synthesized as **27** starting from **37** and **24**. Yield 17% as an oil. ^1^H NMR (CDCl_3_, 400 MHz): δ 0.03 (s, 18H), 0.84–0.88 (t, *J* = 8 Hz, 4H), 1.49–1.53 (t, *J* = 8 Hz, 4H), 4.81 (s, 4H), 7.36–7.43 (m, 2H), 7.48–7.52 (t, *J* = 8 Hz, 2H), 7.54–7.58 (t, *J* = 8 Hz, 2H), 7.64–7.66 (d, *J* = 8 Hz, 2H), 7.76 (s, 1H), 7.86–7.88 (d, *J* = 8 Hz, 2H), 8.09–8.11 (d, *J* = 8 Hz, 2H), 8.48–8.50 (d, *J* = 8 Hz, 1H) ppm. IR: ν 1630 and 2952 cm^–1^.

##### 4-(3-(3,4,5-Trimethoxybenzoyl)-1*H*-indol-1-yl)-*N*,*N*-bis((2-(trimethylsilyl)ethoxy)methyl)benzenesulfonamide (**40**)

It was synthesized as **27** starting from **38** and **24**. Yield 40% as an oil. ^1^H NMR (DMSO-*d*_6_, 400 MHz): δ 0.01 (s, 18H), 0.83–0.87 (m, 4H), 3.48–3.53 (m, 4H), 3.85 (s, 3H), 3.95 (s, 6H), 4.88 (s, 4H), 7.28–7.29 (m, 2H), 7.45–7.52 (m, 2H), 7.72–7.76 (m, 1H), 8.03–8.06 (m, 2H), 8.15–8.18 (m, 2H), 8.39 (s, 1H) and 8.44–8.47 ppm (1H). IR ν 1463 and 2922 cm^–1^.

##### 3-(3-(3,4,5-Trimethoxybenzoyl)-1*H*-indol-1-yl)-*N*,*N*-bis((2-(trimethylsilyl)ethoxy)methyl)benzenesulfonamide (**41**)

It was synthesized as **27** starting from **38** and **24**. Yield 39% as an oil. ^1^H NMR (DMSO-*d*_6_, 400 MHz): δ 0.14 (s, 18H), 0.65 (s, 4H), 3.76 (s, 3H), 3.87 (s, 6H), 4.78 (s, 4H), 7.22 (s, 2H), 7.39 (s, 2H), 7.55 (s, 1H), 7.83–7.85 (m, 1H), 7.96–8.05 (m, 2H), 8.15 (s, 1H), 8.35 (s, 2H) ppm. IR: 1452 and 2892 cm^–1^.

##### 2-(3-(3,4,5-Trimethoxybenzoyl)-1*H*-indol-1-yl)-*N*,*N*-bis((2-(trimethylsilyl)ethoxy)methyl)benzenesulfonamide (**42**)

It was synthesized as **27** starting from **38** and **26**. Yield 61% as an oil. ^1^H NMR (DMSO-*d*_6_, 400 MHz): δ 0.11 (s, 18H), 0.62–0.66 (t, *J* = 8 Hz, 4H), 3.23–3.28 (m, 4H), 3.99–4.15 (m, 4H), 6.98–7.00 (d, *J* = 8 Hz, 1H), 7.22 (s, 2H), 7.27–7.31 (t, *J* = 8 Hz, 1H), 7.34–7.38 (t, *J* = 8 Hz, 1H), 7.65–7.67 (d, *J* = 8 Hz, 1H), 7.79–7.83 (t, *J* = 8 Hz, 1H), 7.87–7.91 (t, *J* = 8 Hz, 1H), 8.19–8.20 (m, 2H), 8.34–8.36 (d, *J* = 8 Hz, 1H) ppm. IR ν 1462 and 2922 cm^–1^.

#### General Procedure for the Preparation of Compounds **24**–**26**

##### Example: 4-Bromo-*N*,*N*-bis((2-(trimethylsilyl)ethoxy)methyl)benzenesulfonamide (**24**)

4-Bromobenzenesulfonamide (1.0 g, 4.24 mmol) in DMF (12 mL) was added dropwise to a suspension of sodium hydride (60% in mineral oil, 408 mg, 9.32 mmol) at 0 °C in DMF (12 mL) under an argon stream. The mixture was allowed to stir at room temperature for 20 min. (2-(Chloromethoxy)ethyl)trimethylsilane (1,47 g, 8.8 mmol) was added dropwise at 0 °C, and the mixture was stirred at room temperature for 30 min. The reaction was quenched on crushed ice and extracted with ethyl acetate. The organic layer was washed with brine, dried on anhydrous sodium sulfate, and filtered. Removal of the solvent gave pure **24** (2.0 g) that was used without further purification. Yield 98% as an oil. ^1^H NMR (CDCl_3_, 400 MHz): δ 0.02 (s, 18H), 0.84 (m, 4H), 3.47 (m, 4H), 4.76 (s, 4H), 7.61 (d, *J* = 8.5 Hz, 2H), 7.76 (d, *J* = 8.5 Hz, 2H) ppm. ^13^C NMR (CDCl_3_, 100 MHz): δ 1.32, 17.96, 65.97, 76.49, 127.65, 129.06, 132.17, 140.54 ppm.

##### 3-Bromo-*N*,*N*-bis((2-(Trimethylsilyl)ethoxy)methyl)benzenesulfonamide (**25**)

It was synthesized as **24** starting from 3-bromobenzenesulfonamide. Yield 97% as an oil. ^1^H NMR (CDCl_3_, 400 MHz): δ 0.02 (s, 18H), 0.83–0.87 (t, *J* = 8 Hz, 4H), 3.46–3.50 (t, *J* = 8 Hz, 4H), 4.76 (s, 4H), 7.33–7.37 (t, *J* = 8 Hz, 1H), 7.65–7.67 (d, *J* = 8 Hz, 1H), 7.81–7.83 (d, *J* = 8 Hz, 1H), 8.07 (s, 1H) ppm.

##### 2-Bromo-*N*,*N*-bis((2-(Trimethylsilyl)ethoxy)methyl)benzenesulfonamide (**26**)

It was synthesized as **24** starting from 2-bromobenzenesulfonamide. Yield 90% as an oil. ^1^H NMR (CDCl_3_, 400 MHz): 0.04 (s, 18H), 0.74–0.78 (t, *J* = 8 Hz, 4H), 3.37–3.41 (t, *J* = 8 Hz, 4H), 4.90 (s, 4H), 7.36–7.40 (t, *J* = 8 Hz, 1H), 7.43–7.47 (t, *J* = 8 Hz, 1H), 7.70–7.72 (d, *J* = 8 Hz, 1H), 8.18–8.20 (d, *J* = 8 Hz, 1H) ppm. ^13^C NMR (CDCl_3_, 100 MHz): δ 1.30, 17.90, 65.84, 76.69, 120.57, 127.74, 132.03, 133.70, 135.52, 140.10 ppm.

### Molecular Modeling

All the docking experiments were performed on a SuperMicro, Intel Xeon Silver powered machine (64 cores in total) running Ubuntu 20.04 LTS. The crystal structures of hCA I (PDB ID: 7Q0D), hCA II (PDB ID: 5E2R), hCA IX (PDB ID: 5FL4), and hCA XII (PDB ID: 6G7A) were downloaded from the PDB (https://www.rcsb.org/) and prepared using Maestro (Schrödinger Release 2022–4: Maestro, Schrödinger, LLC, New York, NY, 2021).^57^ Hydrogen atoms, missing side chains, and missing loops were added; unnecessary side chains, water molecules, and other solvents were removed. The 3D structures of the sulfonamide compounds were protonated at physiological pH and minimized using the OPLS4 force field included in the same software.^58^ AutoDock Vina 1.2.3^59^ was selected as a docking tool owing to its capacity of utilizing the specific zinc-coordination potentials included in the AutoDock4Zn force field.^60^ All prepared proteins were aligned to a chosen reference structure (hCA II, in our case, was used also for numeration of the amino acid residues) using UCSF Chimera,^61^ and the docking center was selected by calculating the geometric center of its cocrystallized ligand. AutoDockTools 1.5.7 was used to visually adjust the grid box sizes to the hCA active site. Docking simulations were run using the AutoDock4^62^ scoring function with an exhaustiveness of 32. Prepared proteins and ligands structures were converted to the PDBQT format using OpenBabel 3.0.1^63,64^ while the grid maps generation and the docking process itself were performed in a batch fashion using a custom Python 2.7.3 script. The docking at the colchicine site of tubulin was performed using 3HKD^65^ for derivative **11** and the 1SA0^66^ structure for derivative **15** and **19**. Proteins were prepared as described for the hCAs. Docking computations were carried out with Plants^67^ using the default setting and a binding site of 12 Å. Reported images were generated by PyMol.^68^

### Biology

#### CA Inhibition Screening Assay

An Applied Photophysics stopped-flow instrument was used for assaying the CA-catalyzed CO_2_ hydration activity.^37^ Phenol red (at a concentration of 0.2 mM) was used as an indicator, working at the maximum absorbance of 557 nm, with 20 mM Hepes (pH 7.5) as buffer and 20 mM sodium sulfate (for maintaining constant ionic strength). The initial rates of the CA-catalyzed CO_2_ hydration reaction were followed for a period of 10–100 s. The CO_2_ concentrations ranged from 1.7 to 17 mM for the determination of the kinetic parameters and inhibition constants. For each inhibitor, at least six traces of the initial 5–10% of the reaction were used for determining the initial velocity. The uncatalyzed rates were determined in the same manner and subtracted from the total observed rates. Stock solutions of the inhibitor (0.1 mM) were prepared in distilled–deionized water, and dilutions up to 0.01 nM were carried out thereafter with distilled–deionized water. Inhibitor and enzyme solutions were preincubated together for 15 min at room temperature prior to assay to allow for the formation of the enzyme–inhibitor complex. The inhibition constants were obtained by nonlinear least-squares methods using the Cheng–Prusoff equation and represent the mean from at least three different determinations. Standard deviations were in the range of ±5–10% of the reported *K*_i_ values. CA isoforms were recombinant enzymes obtained in house as reported previously.^69−71^ The enzyme concentrations in the assay system were as follows: hCA I, 13.2 nM; hCA II, 8.4 nM; hCA IX, 7.9 nM; and hCA XII, 15.2 nM.

#### Tubulin Assembly

The reaction mixtures contained 0.8 M monosodium glutamate (pH 6.6 with HCl in a 2 M stock solution), 10 μM tubulin, 4% (v/v) DMSO, and varying concentrations of compound. Following a 15 min preincubation at 30 °C, samples were chilled on ice, GTP to 0.4 mM was added, and turbidity development was followed at 350 nm in a temperature-controlled recording spectrophotometer for 20 min at 30 °C. The extent of the reaction was measured. Full experimental details were previously described.^72^

#### [^3^H]Colchicine-Binding Assay

The reaction mixtures contained 1.0 μM tubulin, 5.0 μM [^3^H]colchicine, and 5.0 μM inhibitor and were incubated for 10 min at 37 °C. Complete details were described previously.^73^

#### Cell Cultures and Cell Viability Assay

Cell lines were obtained from the American Tissue Culture Collection (ATCC), unless specified otherwise. Cells were grown in Dulbecco’s modified Eagle’s medium (D-MEM) supplemented with 10% fetal bovine serum (FBS) at 37 °C with 5% CO_2_. In all experiments, 300,000 cells were plated in 9 cm^2^ dishes and exposed to test compound dissolved in DMSO (0.1% final concentration) at the indicated concentrations. The methodology for the evaluation of the growth of human MCF-7 breast carcinoma, OVCAR-8, and NCI/ADR-RES cells, obtained from the National Cancer Institute drug screening laboratory, was previously described.^73^ For IC_50_ determinations, OVCAR-8 and NCI/ADR-RES cells were grown in RPMI 1640 medium with 5% FBS, 5% CO_2_ atmosphere at 37 °C, for 96 h. HCT116, SW480, and SW620 cells were grown in DMEM supplemented with 10% FBS and Pen/Strep (15,070–063, GIBCO, Thermo FisherScientific,Waltham, MA, USA). Cell viability was evaluated by sodium (3-[4,5-dimethylthiazol-2-yl]-2,5 diphenyl tetrazolium bromide (MTT)^74^ (HCT116 cells) or 3′-[1-[(phenylamino)-carbonyl]-3,4-tetrazolium]-bis(4-methoxy-6-nitro)benzene-sulfonic acid hydrate (XTT)^75^ (SW480 and SW620 cells). Briefly, cells (range 10–30 × 10^3^ cells/well) were seeded in 96-well microculture plates and then exposed to increasing concentrations of different compounds (range 0–300 μM) for 48 or 72 h. At the end of the treatment, media were removed and incubated at 37 °C in the dark for 4 h in phosphate-buffered saline (PBS) containing 0.2 mg/mL MTT or XTT and PMS (phenazine methosulfate) at a final concentration of 25 μM. Absorbance at 450 nm along with the reference wavelength at 650 nm was measured using a microplate spectrophotometer (Multiskan FC Microplate Photometer, Thermo Scientific, Waltham, MA, USA). The cell growth inhibition rate was calculated utilizing the following formula: inhibition rate (%) = [control OD – (sample OD/control OD)] × 100, where control OD is the absorbance of a negative control, and sample OD is the absorbance of the test sample. The IC_50_ values were determined with GraphPad Prism 5 through constructed dose–response curves. Human MDA-MB-231 TNBC cells (ATCC HTB-26) were grown in DMEM supplemented with 10% FCS, while TNBC cells BT-549 (ATCC HTB-122) were grown in RPMI plus 10% FBS and 1 μg/mL bovine insulin. Cells were kept at low passage, returning to the original frozen stocks every 3–4 months. Hypoxic culture conditions were realized in the presence of 1% O_2_ and 5% CO_2_. The different cell lines were seeded 10,000 cells/well in 48-well plates and treated in 1% FBS with increasing concentrations of compound **15** or reference SLC-0111. After 72 h of incubation at 37 °C with 1% O_2_ and 5% CO_2_, cells were trypsinized, and cell counting was performed with the MACSQuant analyzer (Miltenyi Biotec).^76^ Potential toxicity on healthy cells was evaluated by treating human primary T lymphocytes from two healthy donors with 30 μM **15** or with a control vehicle (DMSO). Healthy donors’ peripheral blood mononuclear cells (PBMCs) were isolated by Lymphoprep (Nycomed) gradient centrifugation. T lymphocytes were negatively selected from PBMCs using magnetic Dynabeads Untouched Human T Cells Kit (Thermo Fisher Scientific) following the manufacturer’s instructions. Apoptotic cell death was evaluated using APC Annexin-V Apoptosis Detection Kit with PI (Thermo Fisher Scientific). Briefly, 1.5 × 10^6^/mL T cells were cultured in 48-well plates, untreated or treated with **15** at 30 μM for 72 h. Cells were then stained using annexin-V/APC and propidium iodide according to the manufacturer’s instruction. Cell populations were acquired using a FACS Canto II flow cytometer (BD Biosciences). Flow cytometric analysis was performed using Flow Jo Flow Cytometric Analysis Software. Human chemosensitive HT29 colon cancer cells were purchased from ATCC (Manassas, VA). These cells were cultured in RPMI 1640 medium supplemented with 10% v/v FBS, 1% v/v penicillin–streptomycin, and 1% v/v l-glutamine. Human HT29/DX cells were generated by stepwise selection in medium with an increasing concentration of DOX, as described previously,^53^ and maintained in culture medium with a final concentration of 200 nM DOX. MDCK and MDCK/P-gp (a gift of Prof. P. Borst, NKI-AVL Institute, Amsterdam, The Netherlands) were grown in DMEM high glucose supplemented with 10% FBS, 1% penicillin, 1% v/v penicillin–streptomycin, and 1% v/v glutamine. All cell lines were authenticated by microsatellite analysis using the PowerPlex kit (Promega Corporation, Madison, WI; last authentication: January 2022). Cells were seeded in 96-well plates. In the first experimental set, cells were incubated for 72 h with DMSO as a solvent or compound **15** at the following concentrations: 0.1 nM, 1 nM, 10 nM, 100 nM, 1 μM, and 10 μM. In the second experimental set, cells were incubated for 72 h with 5 μM DOX alone or with **15** at 1, 10, or 100 nM. Cell viability was evaluated using the WST-1 assay (Sigma-Merck), as per the manufacturer’s instructions, using a Packard EL340 microplate reader (Bio-Tek Instruments, Winooski, VT). The absorbance units of the untreated cells were considered 100%; the absorbance units of the other experimental conditions were expressed as a percentage versus the absorbance units of the untreated cells.

#### Wnt Reporter Assay

Wnt reporter assay was performed as previously described.^77^ HEK293T cells were seeded at 2 × 10^4^ cells/cm^2^ in triplicate in a 24-well plate. The following day, cells were transfected using DreamFect Gold (OZ Biosciences #DG80500) according to the manufacturer’s instructions. 25 ng of the TOP reporter vector (M50 Super 8x TOPFlash Addgene #12456) or 25 ng of the FOP control plasmid (M51 Super 8x FOPFlash Addgene #12457) was transfected in each well in combination with 10 ng of TK Renilla (Promega #E2241) and empty vector (pcDNA3.1) up to 100 ng. After 24 h, cells were incubated with starvation media (Opti-MEM reduced serum medium supplemented with 0.5% FBS, 1% penicillin/streptomycin, 1% sodium pyruvate, and 1% nonessential amino acids) for 8 h. After starvation, Wnt/β-catenin signaling was activated by treating cells with lithium chloride (50 mM) in a starvation medium for 24 h. The following day, cells were treated with increasing concentrations of compound **15** or vehicle (DMSO) for 24 h, and, at the end of the experiment, cells were lysed using Passive Lysis Buffer (Biotium #99912). d-Luciferine (#10101 Biotium) was diluted in the noncommercial luciferase buffer^78^ at a final concentration of 40 μg/mL. Coelenterazine (#S053 Synchem) was diluted in PBS with Ca^2+^ and Mg^2+^ at a final concentration of 0.75 μg/mL. Luminescence was measured using the GloMax Discover Microplate Reader (Promega).

#### ATPase Activity

The P-gp ATPase activity was measured in membrane vesicles, extracted from HT29/DX or MDCK/P-gp cells treated for 3 h with 1, 10, or 100 nM compound **15**, as described previously.^53^ Cells were washed with Ringer’s solution (148.7 mM NaCl, 2.55 mM K_2_HPO_4_, 0.45 mM KH_2_PO_4_, 1.2 mM MgSO_4_; pH 7.4), lysed on crushed ice with lysis buffer (10 mM Hepes/Tris, 5 mM EDTA, 5 mM EGTA, 2 mM dithiothreitol; pH 7.4) supplemented with 2 mM phenylmethylsulfonyl fluoride, 1 mM aprotinin, 10 μg/mL pepstatin, 10 μg/mL leupeptin, and subjected to nitrogen cavitation at 1200 psi for 20 min. Samples were centrifuged at 300*g* for 10 min in the precentrifugation buffer (10 mM Tris/HCl, 25 mM sucrose; pH 7.5), overlaid on a sucrose cushion (10 mM Tris/HCl, 35% w/v sucrose, 1 mM EDTA; pH 7.5), and centrifuged at 14,000*g* for 10 min. The interface was collected, diluted in the centrifugation buffer (10 mM Tris/HCl, 250 mM sucrose; pH 7.5), and subjected to a third centrifugation at 100,000*g* for 45 min. The vesicle pellet was resuspended in 0.5 mL centrifugation buffer and stored at −80 °C until use, after quantification of the protein content. 1 mg of total protein was immunoprecipitated with the anti-P-gp or anti-MRP1 antibody. 100 μg of each immunopurified protein was incubated for 30 min at 37 °C with 50 μL of the reaction mixture (25 mM Tris/HCl, 3 mM ATP, 50 mM KCl, 2.5 mM MgSO_4_, 3 mM dithiothreitol, 0.5 mM EGTA, 2 mM ouabain, 3 mM NaN_3_; pH 7.0). The reaction was stopped by adding 0.2 mL of ice-cold stopping buffer (0.2% w/v ammonium molybdate, 1.3% v/v H_2_SO_4_, 0.9% w/v SDS, 2.3% w/v trichloroacetic acid, and 1% w/v ascorbic acid). After 30 min incubation at room temperature, the absorbance of the phosphate hydrolyzed from ATP was measured at 620 nm, using a Packard EL340 microplate reader. The absorbance was converted into nmoles hydrolyzed phosphate (Pi)/min/mg protein, according to the titration curve previously prepared.

#### Western Blot

Western blot was performed as previously described.^79^ Cells were washed once in ice-cold PBS and lysed in SDS-urea (50 mM Tris-HCl, 2% SDS, 10% glycerol, 10 mM sodium pyrophosphate, 100 mM NaF, 6 M urea, and 10 mM EDTA). Cell lysates were subjected to SDS-polyacrylamide gel electrophoresis and transferred to a nitrocellulose membrane. The membrane was blocked, washed, and incubated with primary antibodies at 37 °C overnight [anti-β-catenin (D10A8) XP mAb #8480 (Cell Signaling), anti-cMyc #9402S (Cell Signaling), anticleaved caspase 3 (Asp175) #9661 (Cell Signaling), and anti-PARP #9542S (Cell Signaling)]. After the membrane was washed, it was incubated with secondary antibodies at 37 °C for 30 min. The membrane was washed again with Tris-buffered saline/Tween-20, and the samples were exposed and imaged using the WesternBright ECL solution (#K-12045-D50 Advansta).

#### Real-Time PCR

Total mRNA was isolated from cells with TriReagent (#R2050–1–200 Zymo Research), and complementary DNA (cDNA) was synthesized by the Sensifast cDNA synthesis kit (#BIO-65054 Bioline). Quantitative PCR was performed with SensiFast Sybr Lo-Rox Mix (#BIO-94020 Bioline), using an Applied Biosystems ViiA 7 Real-Time PCR System instrument (doi: 10.1038/s41389-019-0175-6) and the following amplification primers:

β-actin forward: CACCCTGAAGTACCCCATCGAG;
β-actin reverse: GATCTGGGTCATCTTCTCGCCG;
Fgf20 forward: TAGAGGTGTGGACAGTGGTCTC;
Fgf20 reverse: CTTCAAACTGCTCCCTAAAGATGC;
Sall4 forward: TGCAGCAGTTGGTGGAGAAC;
Sall4 reverse: TCGGTGGCAAATGAGACATTC.

#### Statistical Analysis

All data in the text and figures are provided as means ± SD. The results were analyzed by a one-way analysis of variance (ANOVA), Student’s *t*-test, and Tukey’s test. *p* < 0.05 was considered significant.

## Supporting Information Available

Inhibition of tubulin polymerization (IC_50_ values) by 11, 15, and 19 and references CSA4, 2, and 3: proposed binding mode for derivatives 11, 15, and 19 into the colchicine site on tubulin; effects of 15 on HCT116 cell viability in MTT assay and IC_50_ value determined by nonlinear regression; total and cleaved PARP and Caspase-3 levels in HCT116 cells upon 72 h treatment with 15; dose-dependent viability and intracellular DOX accumulation of 15-treated HT29 and HT29/DX, MDCK, and MDCK/P-gp cells; rate of ATP hydrolysis in HT29/DX and MDCK/P-gp cells upon treatment with increasing concentrations of 15; and UHPLC chromatograms of compounds 11, 15, and 19 and relative area (%) recorded at 254 nm (PDF)
List of molecular formula string spread sheet for all tested compounds and associated biological data (CSV)
Predicted binding mode of 11, 15, and 19 in hCA I (PDB ID: 7Q0D) (PDB)
hCA II (PDB ID: 5E2R) (PDB)
hCA IX (PDB ID: 5FL4) (PDB)
hCA XII (PDB ID: 6G7A) (PDB)

## Supplementary Material

jm3c01424_si_002.csv

jm3c01424_si_003.pdb

jm3c01424_si_004.pdb

jm3c01424_si_005.pdb

jm3c01424_si_006.pdb

## Abbreviations

AAZ: acetazolamide
CA: carbonic anhydrase
CRC: colorectal cancer
CSA4: combretastation A-4
DMSO: dimethyl sulfoxide
DOX: doxorubicin
FBS: fetal bovine serum
5-FU: 5-fluorouracil
PARP: poly(ADP-ribose)polymerase
P-gp: P-glycoprotein
SEM: 2-(trimethylsilyl)ethoxymethyl
TBAF: tetrabutylammonium fluoride
TNBC: triple-negative breast cancer
THF: tetrahydrofuran

## References

[ref1] Supuran C. T. Carbonic anhydrases. Bioorg. Med. Chem. 2013, 21, 1377–1378. 10.1016/j.bmc.2013.02.026. 23452985

[ref2] Alterio V.; Di Fiore A.; D’Ambrosio K.; Supuran C. T.; De Simone G. Multiple binding modes of inhibitors to carbonic anhydrases: how to design specific drugs targeting 15 different isoforms?. Chem. Rev. 2012, 112, 4421–4468. 10.1021/cr200176r. 22607219

[ref3] De Simone G.; Di Fiore A.; Capasso C.; Supuran C. T. The zinc coordination pattern in the η-carbonic anhydrase from Plasmodium falciparum is different from all other carbonic anhydrase genetic families. Bioorg. Med. Chem. Lett. 2015, 25, 1385–1389. 10.1016/j.bmcl.2015.02.046. 25765908

[ref4] Supuran C. T.; Scozzafava A. Carbonic anhydrases as targets for medicinal chemistry. Bioorg. Med. Chem. 2007, 15, 4336–4350. 10.1016/j.bmc.2007.04.020. 17475500

[ref5] McKenna R.. In Carbonic anhydrase: mechanism, regulation, links to disease, industrial applications.; Frost S. C., McKenna R., Eds.; Springer Sci. Bus. Media: Dordrecht, The Netherlands, 2014.

[ref6] Nocentini A.; Supuran C. T. Advances in the structural annotation of human carbonic anhydrases and impact on future drug discovery. Expert Opin. Drug Discovery 2019, 14, 1175–1197. 10.1080/17460441.2019.1651289. 31436118

[ref7] Purkerson J. M.; Schwartz G. J. The role of carbonic anhydrases in renal physiology. Kidney Int. 2007, 71, 103–115. 10.1038/sj.ki.5002020. 17164835

[ref8] Tachibana H.; Gi M.; Kato M.; Yamano S.; Fujioka M.; Kakehashi A.; Hirayama Y.; Koyama Y.; Tamada S.; Nakatani T.; Wanibuchi H. Carbonic anhydrase 2 is a novel invasion-associated factor in urinary bladder cancers. Cancer Sci. 2017, 108, 331–337. 10.1111/cas.13143. 28004470 PMC5378286

[ref9] Supuran C. T.; Scozzafava A.; Mincione F. The development of topically acting carbonic anhydrase inhibitors as antiglaucoma agents. Curr. Pharm. Des. 2008, 14, 649–654. 10.2174/138161208783877866. 18336310

[ref10] Hen N.; Bialer M.; Yagen B.; Maresca A.; Aggarwal M.; Robbins A. H.; McKenna R.; Scozzafava A.; Supuran C. T. Anticonvulsant 4-aminobenzenesulfonamide derivatives with branched-alkylamide moieties: X-ray crystallography and inhibition studies of human carbonic anhydrase isoforms III, VII, and XIV. J. Med. Chem. 2011, 54, 3977–3981. 10.1021/jm200209n. 21506569

[ref11] Pollard A.; Shephard F.; Freed J.; Liddell S.; Chakrabarti L. Mitochondrial proteomic profiling reveals increased carbonic anhydrase II in aging and neurodegeneration. Aging 2016, 8, 2425–2436. 10.18632/aging.101064. 27743511 PMC5115898

[ref12] Ivanov S.; Liao S. Y.; Ivanova A.; Danilkovitch-Miagkova A.; Tarasova N.; Weirich G.; Merrill M. J.; Proescholdt M. A.; Oldfield E. H.; Lee J.; Zavada J.; Waheed A.; Sly W.; Lerman M. I.; Stanbridge E. J. Expression of hypoxia-inducible cell-surface transmembrane carbonic anhydrases in human cancer. Am. J. Pathol. 2001, 158, 905–919. 10.1016/S0002-9440(10)64038-2. 11238039 PMC1850340

[ref13] Becker H. M. Carbonic anhydrase IX and acid transport in cancer. Br. J. Cancer 2020, 122, 157–167. 10.1038/s41416-019-0642-z. 31819195 PMC7051959

[ref14] Parkkila S.; Rajaniemi H.; Parkkila A. K.; Kivela J.; Waheed A.; Pastorekova S.; Pastorek J.; Sly W. S. Carbonic anhydrase inhibitor suppresses invasion of renal cancer cells in vitro. Proc. Natl. Acad. Sci. U.S.A. 2000, 97, 2220–2224. 10.1073/pnas.040554897. 10688890 PMC15781

[ref15] Ilie M. I.; Hofman V.; Ortholan C.; Ammadi R. E.; Bonnetaud C.; Havet K.; Venissac N.; Mouroux J.; Mazure N. M.; Pouyssegur J.; Hofman P. Overexpression of carbonic anhydrase XII in tissues from resectable non-small cell lung cancers is a biomarker of good prognosis. Int. J. Cancer 2011, 128, 1614–1623. 10.1002/ijc.25491. 20521252

[ref16] Yoo C. W.; Nam B. H.; Kim J. Y.; Shin H. J.; Lim H.; Lee S.; Lee S. K.; Lim M. C.; Song Y. J. Carbonic anhydrase XII expression is associated with histologic grade of cervical cancer and superior radiotherapy outcome. Radiat. Oncol. 2010, 5, 101 10.1186/1748-717x-5-101. 21040567 PMC2990746

[ref17] Proescholdt M. A.; Mayer C.; Kubitza M.; Schubert T.; Liao S. Y.; Stanbridge E. J.; Ivanov S.; Oldfield E. H.; Brawanski A.; Merrill M. J. Expression of hypoxia-inducible carbonic anhydrases in brain tumors. Neuro-Oncology 2005, 7, 465–475. 10.1215/S1152851705000025. 16212811 PMC1871734

[ref18] Haapasalo J.; Hilvo M.; Nordfors K.; Haapasalo H.; Parkkila S.; Hyrskyluoto A.; Rantala I.; Waheed A.; Sly W. S.; Pastorekova S.; Pastorek J.; Parkkila A. K. Identification of an alternatively spliced isoform of carbonic anhydrase XII in diffusely infiltrating astrocytic gliomas. Neuro-Oncology 2008, 10, 131–138. 10.1215/15228517-2007-065. 18322268 PMC2613815

[ref19] Hong J. H.; Muhammad E.; Zheng C.; Hershkovitz E.; Alkrinawi S.; Loewenthal N.; Parvari R.; Muallem S. Essential role of carbonic anhydrase XII in secretory gland fluid and HCO3 (−) secretion revealed by disease causing human mutation. J. Physiol. 2015, 593, 5299–5312. 10.1113/JP271378. 26486891 PMC4704518

[ref20] Viikilä P.; Kivelä A. J.; Mustonen H.; Koskensalo S.; Waheed A.; Sly W. S.; Doisy E. A.; Pastorek J.; Pastorekova S.; Parkkila S.; et al. Carbonic anhydrase enzymes II, VII, IX and XII in colorectal carcinomas. World J. Gastroenterol. 2016, 22, 8168–8177. 10.3748/wjg.v22.i36.8168. 27688658 PMC5037085

[ref21] La Regina G.; Coluccia A.; Famiglini V.; Pelliccia S.; Monti L.; Vullo D.; Nuti E.; Alterio V.; De Simone G.; Monti S. M.; Pan P.; Parkkila S.; Supuran C. T.; Rossello A.; Silvestri R. Discovery of 1,1′-biphenyl-4-sulfonamides as a new class of potent and selective carbonic anhydrase XIV inhibitors. J. Med. Chem. 2015, 58, 8564–8572. 10.1021/acs.jmedchem.5b01144. 26497049

[ref22] La Regina G.; Puxeddu M.; Nalli M.; Vullo D.; Gratteri P.; Supuran C. T.; Nocentini A.; Silvestri R. Discovery of new 1,1′-biphenyl-4-sulfonamides as selective subnanomolar human carbonic anhydrase II inhibitors. ACS Med. Chem. Lett. 2020, 11, 633–637. 10.1021/acsmedchemlett.9b00437. 32435363 PMC7236029

[ref23] La Regina G.; Bai R.; Coluccia A.; Famiglini V.; Pelliccia S.; Passacantilli S.; Mazzoccoli C.; Ruggieri V.; Sisinni L.; Bolognesi A.; Rensen W. M.; Miele A.; Nalli M.; Alfonsi R.; Di Marcotullio L.; Gulino A.; Brancale A.; Novellino E.; Dondio G.; Vultaggio S.; Varasi M.; Mercurio C.; Hamel E.; Lavia P.; Silvestri R. New pyrrole derivatives with potent tubulin polymerization inhibiting activity as anticancer agents including Hedgehog-dependent cancer. J. Med. Chem. 2014, 57, 6531–6552. 10.1021/jm500561a. 25025991 PMC4154712

[ref24] La Regina G.; Bai R.; Coluccia A.; Famiglini V.; Passacantilli Sa.; Naccarato V.; Ortar G.; Mazzoccoli C.; Ruggieri V.; Agriesti F.; Piccoli C.; Tataranni T.; Nalli M.; Brancale A.; Vultaggio S.; Mercurio C.; Varasi M.; Saponaro C.; Sergio Sa.; Maffia M.; Coluccia A.; Hamel E.; Silvestri R. 3-Aroyl-1,4-diarylpyrroles inhibit chronic myeloid leukemia cell growth through an interaction with tubulin. ACS Med. Chem. Lett. 2017, 8, 521–526. 10.1021/acsmedchemlett.7b00022. 28523104 PMC5430391

[ref25] De Martino G.; Edler M. C.; La Regina G.; Coluccia A.; Barbera M. C.; Barrow D.; Nicholson R. I.; Chiosis G.; Brancale A.; Hamel E.; Artico M.; Silvestri R. New Arylthioindoles: Potent Inhibitors of Tubulin Polymerization. 2. Structure–Activity Relationships and Molecular Modeling Studies. J. Med. Chem. 2006, 49, 947–954. 10.1021/jm050809s. 16451061

[ref26] Guo S.; Zhen Y.; Guo M.; Zhang L.; Zhou G. Design, synthesis and antiproliferative evaluation of novel sulfanilamide-1,2,3-triazole derivatives as tubulin polymerization inhibitors. Invest. New Drugs 2018, 36, 1147–1157. 10.1007/s10637-018-0632-7. 30019099

[ref27] Matsue T.; Gi M.; Shiota M.; Tachibana H.; Suzuki S.; Fujioka M.; Kakehashi A.; Yamamoto T.; Kato M.; Uchida J.; Wanibuchi H. The carbonic anhydrase inhibitor acetazolamide inhibits urinary bladder cancers via suppression of β-catenin signaling. Cancer Sci. 2022, 113, 2642–2653. 10.1111/cas.15467. 35723039 PMC9357660

[ref28] McConkey D. J.; Choi W.; Marquis L.; Martin F.; Williams M. B.; Shah J.; Svatek R.; Das A.; Adam L.; Kamat A.; Siefker-Radtke A.; Dinney C. Role of epithelial-to-mesenchymal transition (EMT) in drug sensitivity and metastasis in bladder cancer. Cancer Metastasis Rev. 2009, 28, 335–344. 10.1007/s10555-009-9194-7. 20012924 PMC5915353

[ref29] Di Magno L.; Di Pastena F.; Puxeddu M.; La Regina G.; Coluccia A.; Ciogli A.; Manetto S.; Maroder M.; Canettieri G.; Silvestri R.; Nalli M. Sulfonamide inhibitors of β-catenin signaling as anticancer agents with different output on c-MYC. ChemMedChem 2020, 15, 2264–2268. 10.1002/cmdc.202000594. 32946182

[ref30] Fang L.; Zhu Q.; Neuenschwander M.; Specker E.; Wulf-Goldenberg A.; Weis W. I.; von Kries J. P.; Birchmeier W. A small molecule antagonist of the β-catenin/TCF4 interaction blocks the self-renewal of cancer stem cells and suppresses tumorigenesis. Cancer Res. 2016, 76, 891–901. 10.1158/0008-5472.CAN-15-1519. 26645562

[ref31] Kopecka J.; Campia I.; Jacobs A.; Frei A. P.; Ghigo D.; Wollscheid B.; Riganti C. Carbonic anhydrase XII is a new therapeutic target to overcome chemoresistance in cancer cells. Oncotarget 2015, 6, 6776–6793. 10.18632/oncotarget.2882. 25686827 PMC4466649

[ref32] Kopecka J.; Rankin G. M.; Salaroglio I. C.; Poulsen S. A.; Riganti C. P-glycoprotein-mediated chemoresistance is reversed by carbonic anhydrase XII inhibitors. Oncotarget 2016, 7, 85861–85875. 10.18632/oncotarget.13040. 27811376 PMC5349880

[ref33] Tardia P.; Stefanachi A.; Niso M.; Stolfa D. A.; Mangiatordi G. F.; Alberga D.; Nicolotti O.; Lattanzi G.; Carotti A.; Leonetti F.; Perrone R.; Berardi F.; Azzariti A.; Colabufo N. A.; Cellamare S. Trimethoxybenzanilide-based P-glycoprotein modulators: an interesting case of lipophilicity tuning by intramolecular hydrogen bonding. J. Med. Chem. 2014, 57, 6403–6418. 10.1021/jm500697c. 25093931

[ref34] Puxeddu M.; Wu J.; Bai R.; D’Ambrosio M.; Nalli M.; Coluccia A.; Manetto S.; Ciogli A.; Masci D.; Urbani A.; Fionda C.; Coni S.; Bordone R.; Canettieri G.; Bigogno C.; Dondio G.; Hamel E.; Liu T.; Silvestri R.; La Regina G. Induction of ferroptosis in glioblastoma and ovarian cancers by a new pyrrole tubulin assembly inhibitor. J. Med. Chem. 2022, 65, 15805–15818. 10.1021/acs.jmedchem.2c01457. 36395526 PMC9743090

[ref35] Artico M.; Di Santo R.; Costi R.; Massa S.; Retico A.; Artico M.; Apuzzo G.; Simonetti G.; Strippoli V. Antifungal agents. 9. 3-Aryl-4-[alpha-(1*H*-imidazole-1-yl)arylmethyl]pyrroles: a new class of potent anti-Candida agents. J. Med. Chem. 1995, 38, 4223–4233. 10.1021/jm00021a011. 7473549

[ref36] La Regina G.; Sarkar T.; Bai R.; Edler M. C.; Saletti R.; Coluccia A.; Piscitelli F.; Minelli L.; Gatti V.; Mazzoccoli C.; Palermo V.; Mazzoni C.; Falcone C.; Scovassi A. I.; Giansanti V.; Campiglia P.; Porta A.; Maresca B.; Hamel E.; Brancale A.; Novellino E.; Silvestri R. New arylthioindoles and related bioisosteres at the sulfur bridging group. 4. Synthesis, tubulin polymerization, cell growth inhibition, and molecular modeling studies. J. Med. Chem. 2009, 52, 7512–7527. 10.1021/jm900016t. 19601594 PMC2797757

[ref37] Khalifah R. G. The Carbon Dioxide Hydration Activity of Carbonic Anhydrase. J. Biol. Chem. 1971, 246, 2561–2573. 10.1016/S0021-9258(18)62326-9. 4994926

[ref38] Farzam K.; Abdullah M. Acetazolamide. NIH, National Library of Medicine. Last Update: July 10, 2022. https://www.ncbi.nlm.nih.gov/books/NBK532282/ (accessed Jan 29, 2023).

[ref39] Bissantz C.; Kuhn B.; Stahl M. A medicinal chemist’s guide to molecular interactions. J. Med. Chem. 2010, 53, 5061–5084. 10.1021/jm100112j. 20345171 PMC2905122

[ref40] Arnold A.; Tronser M.; Sers C.; Ahadova A.; Endris V.; Mamlouk S.; Horst D.; Möbs M.; Bischoff P.; Kloor M.; Bläker H. The majority of β-catenin mutations in colorectal cancer is homozygous. BMC Cancer 2020, 20, 1038 10.1186/s12885-020-07537-2. 33115416 PMC7594410

[ref41] Korinek V.; Barker N.; Morin P.; van Wichen D.; de Weger R.; Kinzler K.; Vogelstein B.; Clevers H. Constitutive transcriptional activation by a β-catenin-Tcf complex in APC–/– colon carcinoma. Science 1997, 275, 1784–1787. 10.1126/science.275.5307.1784. 9065401

[ref42] Morin P.; Sparks A.; Korinek V.; Barker N.; Clevers H.; Vogelstein B.; Kinzler K. Activation of β-catenin-Tcf signaling in colon cancer by mutations in β-catenin or APC. Science 1997, 275, 1787–1790. 10.1126/science.275.5307.1787. 9065402

[ref43] La Regina G.; Bai R.; Rensen W.; Coluccia A.; Piscitelli F.; Gatti V.; Bolognesi A.; Lavecchia A.; Granata I.; Porta A.; Maresca B.; Soriani A.; Iannitto M. L.; Mariani M.; Santoni A.; Brancale A.; Ferlini C.; Dondio G.; Varasi M.; Mercurio C.; Hamel E.; Lavia P.; Novellino E.; Silvestri R. Design and synthesis of 2-heterocyclyl-3-arylthio-1*H*-indoles as potent tubulin polymerization and cell growth inhibitors with improved metabolic stability. J. Med. Chem. 2011, 54, 8394–8406. 10.1021/jm2012886. 22044164 PMC3261769

[ref44] McDonald P. C.; Chia S.; Bedard P. L.; Chu Q.; Lyle M.; Tang L.; Singh M.; Zhang Z.; Supuran C. T.; Renouf D. J.; Dedhar S. A Phase 1 study of SLC-0111, a novel inhibitor of carbonic anhydrase IX, in patients with advanced solid tumors. Am. J. Clin. Oncol. 2020, 43, 484–490. 10.1097/COC.0000000000000691. 32251122 PMC7323835

[ref45] Soldani C.; Scovassi A. I. Poly(ADP-ribose) polymerase-1 cleavage during apoptosis: an update. Apoptosis 2002, 7, 321–328. 10.1023/A:1016119328968. 12101391

[ref46] Crowley L. C.; Waterhouse N. J. Detecting cleaved caspase-3 in apoptotic cells by flow cytometry. Cold Spring Harb. Protoc. 2016, 2016 (11), pdb.prot087312 10.1101/pdb.prot087312. 27803251

[ref47] Zhang M.; Du H.; Wang L.; Yue Y.; Zhang P.; Huang Z.; Lv W.; Ma J.; Shao Q.; Ma M.; Liang X.; Yang T.; Wang W.; Zeng J.; Chen G.; Wang X.; Fan J. Thymoquinone suppresses invasion and metastasis in bladder cancer cells by reversing EMT through the Wnt/β-catenin signaling pathway. Chem. Biol. Interact. 2020, 320, 109022 10.1016/j.cbi.2020.109022. 32112862

[ref48] Riganti C.; Kopecka J.; Panada E.; Barak S.; Rubinstein M. The role of C/EBP-β LIP in multidrug resistance. J. Natl. Cancer Inst. 2015, 107, djv046 10.1093/jnci/djv046. 25766403

[ref49] Braconi L.; Teodori E.; Riganti C.; Coronnello M.; Nocentini A.; Bartolucci G.; Pallecchi M.; Contino M.; Manetti D.; Romanelli M. N.; Supuran C. T.; Dei S. New dual P-glycoprotein (P-gp) and human carbonic anhydrase XII (hCA XII) inhibitors as multidrug resistance (MDR) reversers in cancer cells. J. Med. Chem. 2022, 65, 14655–14672. 10.1021/acs.jmedchem.2c01175. 36269278 PMC9661477

[ref50] Gazzano E.; Chegaev K.; Rolando B.; Blangetti M.; Annaratone L.; Ghigo D.; Fruttero R.; Riganti C. Overcoming multidrug resistance by targeting mitochondria with NO-donating doxorubicins. Bioorg. Med. Chem. 2016, 24, 967–975. 10.1016/j.bmc.2016.01.021. 26822567

[ref51] Pastan I.; Gottesman M. M.; Ueda K.; Lovelace E.; Rutherford A. V.; Willingham M. C. A retrovirus carrying an MDR1 cDNA confers multidrug resistance and polarized expression of P-glycoprotein in MDCK cells. Proc. Natl. Acad. Sci. U.S.A. 1988, 85, 4486–4490. 10.1073/pnas.85.12.4486. 2898143 PMC280455

[ref52] Arnason T.; Harkness T. Development, maintenance, and reversal of multiple drug resistance: at the crossroads of TFPI1, ABC transporters, and HIF1. Cancers 2015, 7, 2063–2082. 10.3390/cancers7040877. 26501324 PMC4695877

[ref53] Kopecka J.; Salzano G.; Campia I.; Lusa S.; Ghigo D.; De Rosa G.; Riganti C. Insights in the chemical components of liposomes responsible for P-glycoprotein inhibition. Nanomedicine 2014, 10, 77–87. 10.1016/j.nano.2013.06.013. 23850894

[ref54] Riganti C.; Gazzano E.; Gulino G. R.; Volante M.; Ghigo D.; Kopecka J. Two repeated low doses of doxorubicin are more effective than a single high dose against tumors overexpressing P-glycoprotein. Cancer Lett. 2015, 360, 219–226. 10.1016/j.canlet.2015.02.008. 25681670

[ref55] Lipinski C. A.; Lombardo F.; Dominy B. W.; Feeney P. J. Experimental and computational approaches to estimate solubility and permeability in drug discovery and development settings. Adv. Drug Delivery Rev. 1997, 23, 3–25. 10.1016/s0169-409x(96)00423-1. 11259830

[ref56] Veber D. F.; Johnson S. R.; Cheng H. Y.; Smith B. R.; Ward K. W.; Kopple K. D. Molecular properties that influence the oral bioavailability of drug candidates. J. Med. Chem. 2002, 45, 2615–2623. 10.1021/jm020017n. 12036371

[ref57] Madhavi Sastry G.; Adzhigirey M.; Day T.; Annabhimoju R.; Sherman W. Protein and ligand preparation: parameters, protocols, and influence on virtual screening enrichments. J. Comput. Aided Mol. Des. 2013, 27, 221–234. 10.1007/s10822-013-9644-8. 23579614

[ref58] Lu C.; Wu C.; Ghoreishi D.; Chen W.; Wang L.; Damm W.; Ross G. A.; Dahlgren M. K.; Russell E.; Von Bargen C. D.; Abel R.; Friesner R. A.; Harder E. D. OPLS4: improving force field accuracy on challenging regimes of chemical space. J. Chem. Theory Comput. 2021, 17, 4291–4300. 10.1021/acs.jctc.1c00302. 34096718

[ref59] Eberhardt J.; Santos-Martins D.; Tillack A. F.; Forli S. AutoDock Vina 1.2.0: new docking methods, expanded force field, and python bindings. J. Chem. Inf. Model. 2021, 61, 3891–3898. 10.1021/acs.jcim.1c00203. 34278794 PMC10683950

[ref60] Santos-Martins D.; Forli S.; Ramos M. J.; Olson A. J. AutoDock4(Zn): an improved AutoDock force field for small-molecule docking to zinc metalloproteins. J. Chem. Inf. Model. 2014, 54, 2371–2379. 10.1021/ci500209e. 24931227 PMC4144784

[ref61] Pettersen E. F.; Goddard T. D.; Huang C. C.; Couch G. S.; Greenblatt D. M.; Meng E. C.; Ferrin T. E. UCSF Chimera-a visualization system for exploratory research and analysis. J. Comput. Chem. 2004, 25, 1605–1612. 10.1002/jcc.20084. 15264254

[ref62] Morris G. M.; Huey R.; Lindstrom W.; Sanner M. F.; Belew R. K.; Goodsell D. S.; Olson A. J. AutoDock4 and AutoDockTools4: automated docking with selective receptor flexibility. J. Comput. Chem. 2009, 30, 2785–2791. 10.1002/jcc.21256. 19399780 PMC2760638

[ref63] Huey R.; Morris G. M.; Olson A. J.; Goodsell D. S. A semiempirical free energy force field with charge-based desolvation. J. Comput. Chem. 2007, 28, 1145–1152. 10.1002/jcc.20634. 17274016

[ref64] O’Boyle N. M.; Banck M.; James C. A.; Morley C.; Vandermeersch T.; Hutchison G. R. Open Babel: an open chemical toolbox. J. Cheminf. 2011, 3, 33 10.1186/1758-2946-3-33. PMC3198950 21982300

[ref65] Dorleans A.; Gigant B.; Ravelli R. B. G.; Mailliet P.; Mikol V.; Knossow M. Variations in the colchicine-binding domain provide insight into the structural switch of tubulin. Proc. Natl. Acad. Sci. U.S.A. 2009, 106, 13775–13779. 10.1073/pnas.0904223106. 19666559 PMC2728970

[ref66] Ravelli R. B.; Gigant B.; Curmi P. A.; Jourdain I.; Lachkar S.; Sobel A.; Knossow M. Insight into tubulin regulation from a complex with colchicine and a stathmin-like domain. Nature 2004, 428, 198–202. 10.1038/nature02393. 15014504

[ref67] Korb O.; Stutzle T.; Exner T. E. PLANTS: application of ant colony optimization to structure-based drug design. In Ant Colony Optimization and Swarm Intelligence, Proceedings of the 5th International Workshop, ANTS; Dorigo M.; Gambardella L. M.; Birattari M.; Martinoli A.; Poli R.; Stutzle T., Eds.; Lecture Notes in Computer Science, Series 4150; Springer: Berlin, 2006; pp 247–258.

[ref68] The PyMol Molecular Graphics System, Version 2.0; Schrödinger, LLC: 2015.

[ref69] Vitale R. M.; Alterio V.; Innocenti A.; Winum J. Y.; Monti S. M.; De Simone G.; Supuran C. T. Carbonic anhydrase inhibitors. Comparison of aliphatic sulfamate/bis-sulfamate adducts with isozymes II and IX as a platform for designing tight-binding, more isoform-selective inhibitors. J. Med. Chem. 2009, 52, 5990–5998. 10.1021/jm900641r. 19731956

[ref70] D’Ambrosio K.; Smaine F. Z.; Carta F.; De Simone G.; Winum J.-Y.; Supuran C. T. Development of potent carbonic anhydrase inhibitors incorporating both sulfonamide and sulfamide groups. J. Med. Chem. 2012, 55, 6776–6783. 10.1021/jm300818k. 22775345

[ref71] Pacchiano F.; Carta F.; McDonald P. C.; Lou Y.; Vullo D.; Scozzafava A.; Dedhar S.; Supuran C. T. Ureido-substituted benzenesulfonamides potently inhibit carbonic anhydrase IX and show antimetastatic activity in a model of breast cancer metastasis. J. Med. Chem. 2011, 54, 1896–1902. 10.1021/jm101541x. 21361354

[ref72] Hamel E. Evaluation of antimitotic agents by quantitative comparisons of their effects on the polymerization of purified tubulin. Cell Biochem. Biophys. 2003, 38, 1–22. 10.1385/CBB:38:1:1. 12663938

[ref73] Verdier-Pinard P.; Lai J.-Y.; Yoo H.-D.; Yu J.; Marquez B.; Nagle D. G.; Nambu M.; White J. D.; Falck J. R.; Gerwick W. H.; Day B. W.; Hamel E. Structure-activity analysis of the interaction of curacin A, the potent colchicine site antimitotic agent, with tubulin and effects of analogs on the growth of MCF-7 breast cancer cells. Mol. Pharmacol. 1998, 53, 62–76. 10.1124/mol.53.1.62. 9443933

[ref74] Van Meerloo J.; Kaspers G. J. L.; Cloos J. Cell Sensitivity Assays: The MTT Assay. Methods Mol. Biol. 2011, 731, 237–245. 10.1007/978-1-61779-080-5_20. 21516412

[ref75] Roehm N. W.; Rodgers G. H.; Hatfield S. M.; Glasebrook A. L. An improved colorimetric assay for cell proliferation and viability utilizing the tetrazolium salt XTT. J. Immunol. Methods 1991, 142, 257–265. 10.1016/0022-1759(91)90114-u. 1919029

[ref76] Giacomini A.; Taranto S.; Rezzola S.; Matarazzo S.; Grillo E.; Bugatti M.; Scotuzzi A.; Guerra J.; Di Trani M.; Presta M.; Ronca R. Inhibition of the FGF/FGFR system induces apoptosis in lung cancer cells via c-myc downregulation and oxidative stress. Int. J. Mol. Sci. 2020, 21, 9376 10.3390/ijms21249376. 33317057 PMC7763353

[ref77] Coluccia A.; La Regina G.; Naccarato V.; Nalli M.; Orlando V.; Biagioni S.; De Angelis M. L.; Baiocchi M.; Gautier C.; Gianni S.; Di Pastena F.; Di Magno L.; Canettieri G.; Coluccia A. M. L.; Silvestri R. Drug design and synthesis of first in class PDZ1 targeting NHERF1 inhibitors as anticancer agents. ACS Med. Chem. Lett. 2019, 10, 499–503. 10.1021/acsmedchemlett.8b00532. 30996786 PMC6466550

[ref78] Dyer B. W.; Ferrer F. A.; Klinedinst D. K.; Rodriguez R. A noncommercial dual luciferase enzyme assay system for reporter gene analysis. Anal. Biochem. 2000, 282, 158–161. 10.1006/abio.2000.4605. 10860516

[ref79] Coni S.; Bordone R.; Ivy D. M.; Yurtsever Z. N.; Di Magno L.; D’Amico R.; Cesaro B.; Fatica A.; Belardinilli F.; Bufalieri F.; Maroder M.; De Smaele E.; Di Marcotullio L.; Giannini G.; Agostinelli E.; Canettieri G. Combined inhibition of polyamine metabolism and eIF5A hypusination suppresses colorectal cancer growth through a converging effect on MYC translation. Cancer Lett. 2023, 559, 216120 10.1016/j.canlet.2023.216120. 36893894

