# Supplementary material for: 4-(3-Phenyl-4-(3,4,5-trimethoxybenzoyl)-1H-pyrrol-1-yl)benzenesulfonamide, a Novel Carbonic Anhydrase and Wnt/β-Catenin Signaling Pathway Dual-Targeting Inhibitor with Potent Activity against Multidrug Resistant Cancer Cells
Source: J Med Chem. 2023 Oct 30;66(21):14824–42. doi: 10.1021/acs.jmedchem.3c01424 (PMC10641813; doi:10.1021/acs.jmedchem.3c01424)
Supplement: Supplementary file 1 — jm3c01424_si_001.pdf [file jm3c01424_si_001.pdf]

Supporting Information

4-(3-Phenyl-4-(3,4,5-trimethoxybenzoyl)-1*H*-pyrrol-1-yl)benzenesulfonamide, a Novel Carbonic Anhydrase and Wnt/β-catenin Signaling Pathway Dual Targeting Inhibitor with Potent Activity Against Multi-Drug Resistant Cancer Cells

Domiziana Masci,[+,a] Michela Puxeddu,[+,b] Laura Di Magno[+,c] Michele D'Ambrosio[b] Anastasia Parisi,[b] Marianna Nalli,[b] Ruoli Bai,[d] Antonio Coluccia,[b] Pietro Sciò,[b] Viviana Orlando,[e] Sara D'Angelo,[e] Stefano Biagioni,[e] Andrea Urbani,[a] Ernest Hamel,[d] Alessio Nocentini,[f] Serena Filiberti,[g] Marta Turati,[g] Roberto Ronca,[g] Joanna Kopecka,[h] Chiara Riganti,[h] Cinzia Fionda,[c] Rosa Bordone,[c] Giorgia Della Rocca,[c] Gianluca Canettieri,[*,c] Claudiu T. Supuran,[f] Romano Silvestri,[*,b] Giuseppe La Regina,[b]

[a] Department of Basic Biotechnological Sciences, Intensivological and Perioperative Clinics, Catholic University of the Sacred Heart, Largo Francesco Vito 1, 00168 Rome, Italy

[b] Laboratory affiliated with the Institute Pasteur Italy – Cenci Bolognetti Foundation, Department of Drug Chemistry and Technologies, Sapienza University of Rome, Piazzale Aldo Moro 5, 00185 Roma, Italy

[c] Laboratory affiliated to Istituto Pasteur Italia – Fondazione Cenci Bolognetti, Department of Molecular Medicine, Sapienza University of Rome, Viale Regina Elena 291, 00161 Rome, Italy

[d] Molecular Pharmacology Branch, Developmental Therapeutics Program, Division of Cancer Treatment and Diagnosis, Frederick National Laboratory for Cancer Research, National Cancer Institute, National Institutes of Health, Frederick, Maryland 21702, United States

[e] Department of Biology and Biotechnologies "Charles Darwin", Sapienza University of Rome, Piazzale Aldo Moro 5, 00185 Rome, Italy

[f] Dipartimento Neurofarba, Sezione di Scienze Farmaceutiche e Nutraceutiche, Università degli Studi di Firenze, Via Ugo Schiff 6, I-50019 Sesto Fiorentino, Firenze, Italy

[g] Experimental Oncology and Immunology Unit, Department of Molecular and Translational Medicine, University of Brescia, via Branze 39, 25123 Brescia, Italy

[h] Oncological Pharmacology Unit, Department of Oncology and Molecular Biotecnology Center "Guido Tarone", via Nizza 44, 10126 Torino, Italy

Corresponding Author's email address. Gianluca Canettieri, gianluca.canettieri@uniroma1.it: Romano Silvestri, romano.silvestri@uniroma1.it.

Contents of SI

Table S1. Inhibition of Tubulin Polymerization by Compounds **11**, **15** and **19.**

Figure S1. Proposed binding mode for derivatives **11**, **15** and **19** into the colchicine site on tubulin. **11** and **15** are compared to the respective parent compounds **2** and **3**.

Figure S2. Effects of compound **15** on viability of HCT116 cells.

Figure S3. Total and cleaved PARP levels in HCT116 cells upon 72 h treatment with **15**.
Figure S4. Total and cleaved Caspase-3 levels in HCT116 cells upon 72 h treatment with **15.**
Figure S5. Dose-dependent viability of HT29 and HT29/DX, MDCK and MDCK/P-gp cells treated with **15**.
Figure S6. Intracellular accumulation of DOX in HT29, HT29/DX, MDCK and MDCK/P-gp cells treated with **15**.
Table S2. ATP hydrolysis by**15** in HT29/DX and MDCK-Pgp cells.
Figure S7. HPLC chromatograms of compounds **11**.
Figure S8. HPLC chromatograms of compounds **15**.
Figure S9. HPLC chromatograms of compounds **19**.
Table S3. Relative area (%) recorded at 254 nm for compounds **11, 15** and **19.**

Table S1. Inhibition of Tubulin Polymerization by Compounds 11, 15 and 19 and Combretastatin A-4 as Reference Compound.[a]

Compd	Tubulin $IC_{50} \pm SD$ (μM)[b]
**11**	>20[c]
**15**	>20[d]
**19**	>20[d]
CSA4	0.75 ± 0.06
**2**	1.5 ± 0.2[e]
**3**	1.1 ± 0.1[f]

[a]Experiments were performed in duplicate or triplicate. [b]Tubulin was at 10 μM in the assembly assay. [c]No activity. [d]Partial activity. [e]Ref. 23. [f]Ref. 24.

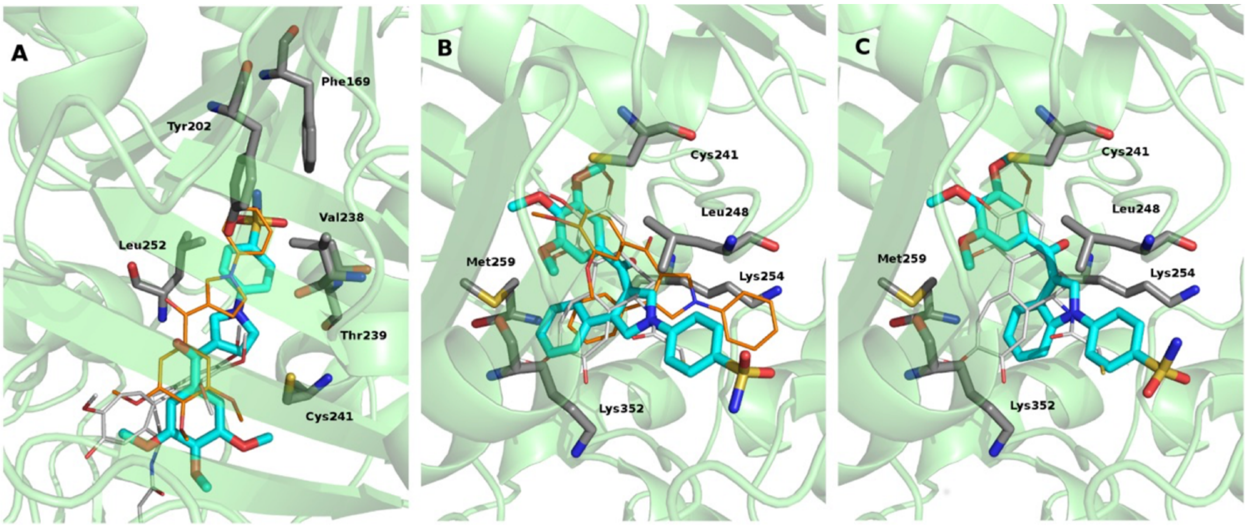

Figure S1. Proposed binding mode for derivatives **11** (panel A), **15** (panel B) and **19** (panel C) all shwn as cyan sticks, into the colchicine site on tubulin. The parent compounds **2** (panel A) and **3** (panel B) are shown as orange lines. The residues involved in the interactions are reported are shown as grey sticks; tubulin is shown as a green cartoon.

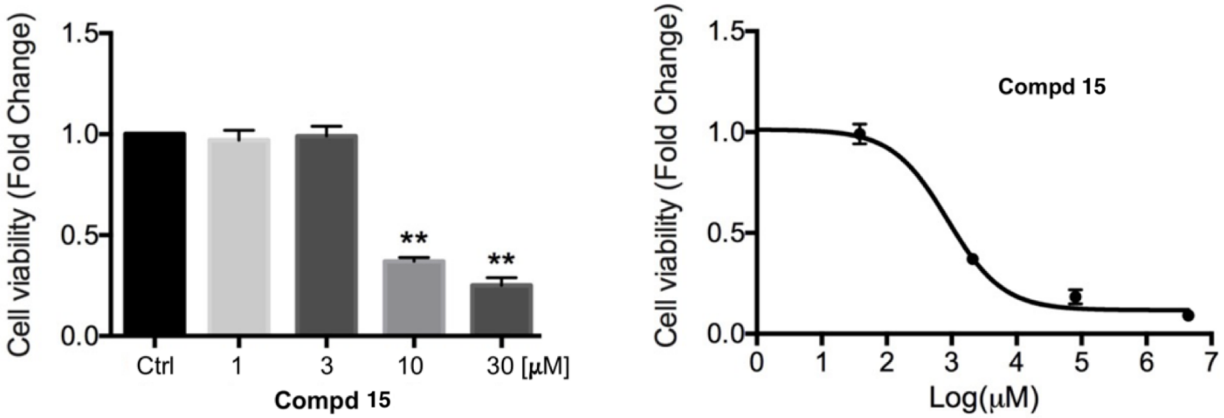

Figure S2. Effects of compound **15** on viability of HCT116 cells. The cells were treated with increasing concentrations of **15** for 72 h. Left panel: cell viability was analyzed by the MTT assay; right panel, IC_{50} value was determined by non-linear regression.

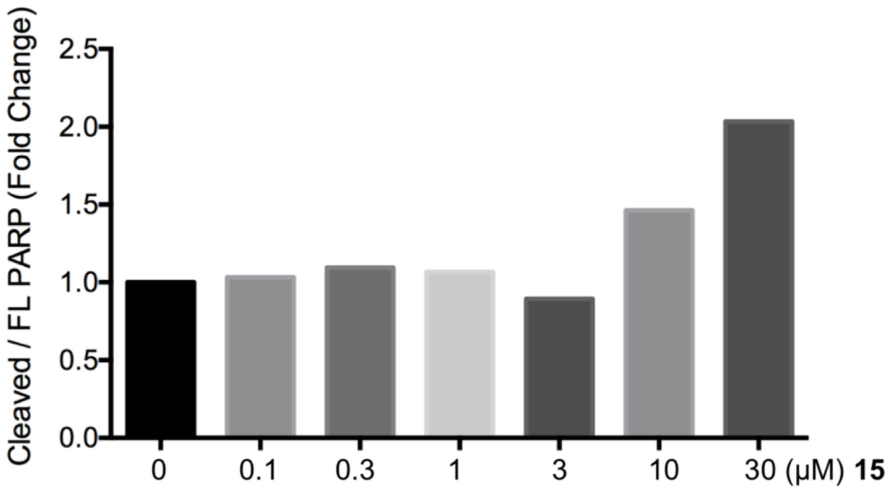

Figure S3. Total and cleaved PARP levels in HCT116 cells upon 72 h treatment with increasing concentrations of compound **15**. Densitometric analysis of western blot results are shown.

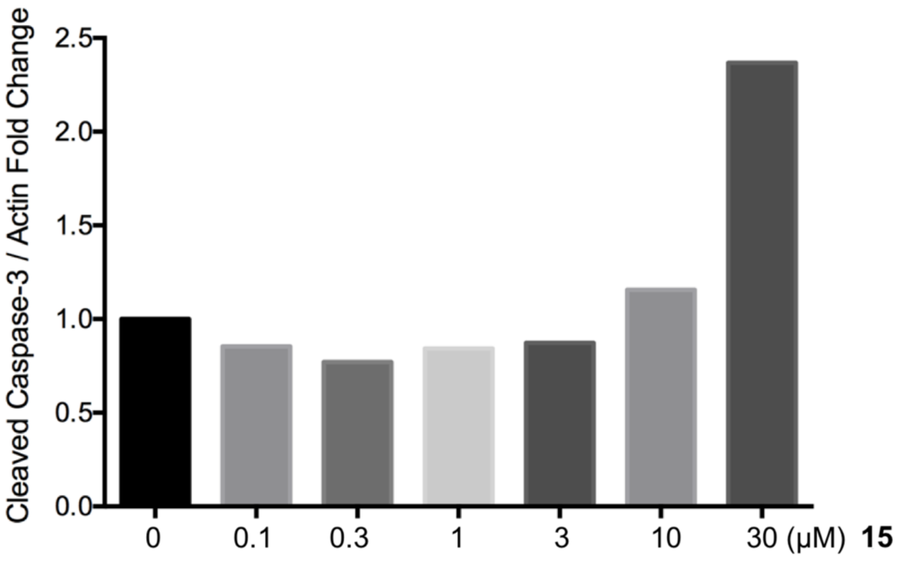

Figure S4. Total and cleaved Caspase-3 levels in HCT116 cells upon 72 h treatment with increasing concentrations of compound **15**. Densitometric analysis of western blot results are shown.

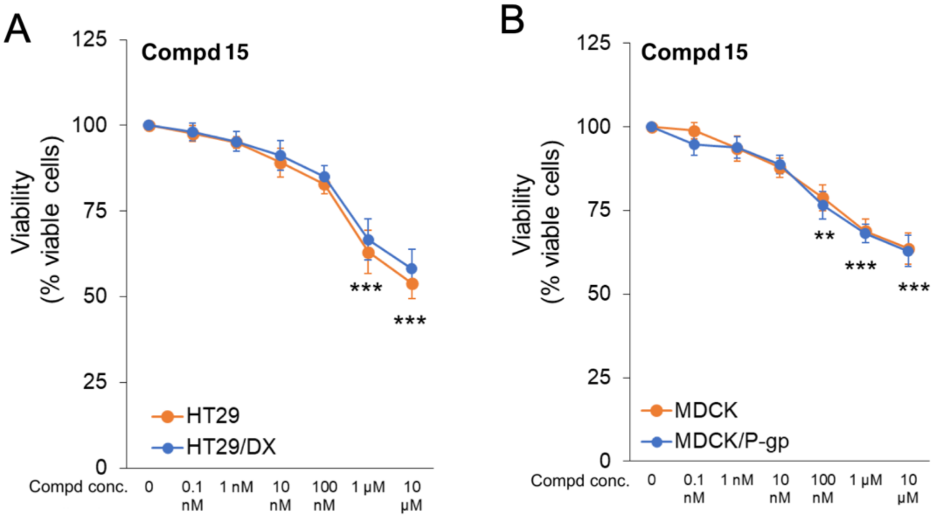

Figure S5. Dose-dependent viability of HT29 and HT29/DX (A), MDCK and MDCK/P-gp (B) cells, incubated with increasing concentrations (0-10 µM) of **15** for 72 h, measured with a spectrophotometric assay. Data are means ± SD (n = 3). **p<0.01, ***p<0.001: versus untreated cells ("0").

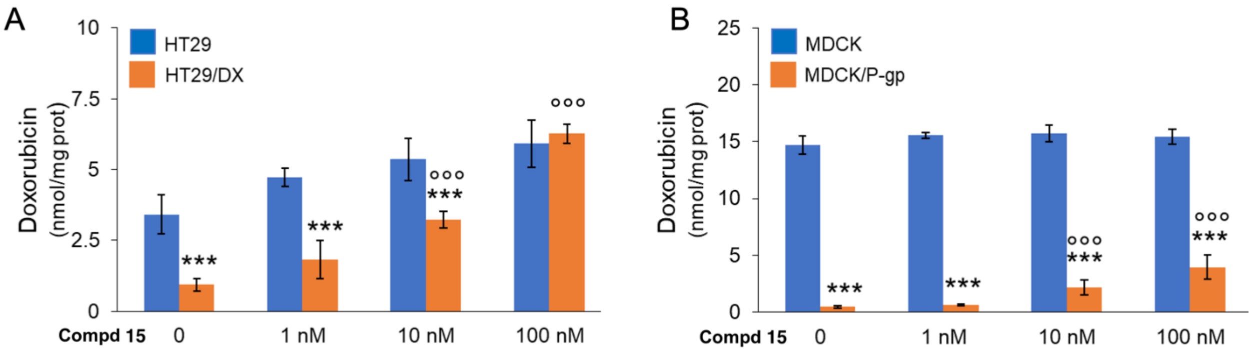

Figure S6. Intracellular accumulation of DOX in HT29 and HT29/DX cells (A), MDCK and MDCK/P-gp (B), incubated 3 h with 5 µM DOX, in the absence ("0") or presence of compound **15** at 1, 10 and 100 nM. The intracellular drug retention was measured spectrofluorimetrically. Data are means ± SD (n=3)., ***p<0.001: versus HT29 or MDCK cells treated with DOX alone; °°p<0.01, °°°p<0.001: versus HT29/DX or MDCK/P-gp cells treated with DOX alone.

Table S2. Rate of ATP hydrolysis in HT29/DX and MDCK-Pgp cells upon treatmet with increasing concentrations of compound 15.[a]

	HT29/DX			MDCK-Pgp		
**Pgp activity**[b]	**I exp**[c]	**II exp**	**III exp**	**I exp**	**II exp**	**III exp**
Ctrl[d]	1.2	1.0	1.4	6.7	6.5	6.2
	0.9	0.8	1.0	5.6	6.5	7.3
	1.0	1.3	1.1	4.5	7.1	5.8
**15** 1 nM	0.7	0.8	0.9	5.2	5.6	5.6
	0.8	0.6	1.0	5.4	5.5	5.9
	0.7	0.6	0.8	5.1	5.4	5.3
**15** 10 nM	0.4	0.6	0.5	3.4	3.2	3.4
	0.5	0.4	0.6	3.2	3.4	3.0
	0.6	0.4	0.7	3.9	3.2	3.8
**15** 100 nM	0.1	0.2	0.3	2.1	2.4	2.3
	0.3	0.3	0.2	2.4	1.6	1.8
	0.2	0.3	0.3	1.7	1.8	1.5

[a]The proteins from HT29/DX and MDCK/P-gp cells were immunopurified and the absorbance of the phosphate hydrolyzed from ATP was measured at 620 nm as previously reported (Ref. 1S). [b]P-gp activity is expressed as nmol Pi × min^{-1} × mg $protein^{-1}$. [c]Experiments were performed in triplicate. [d]ctrl, no compound.

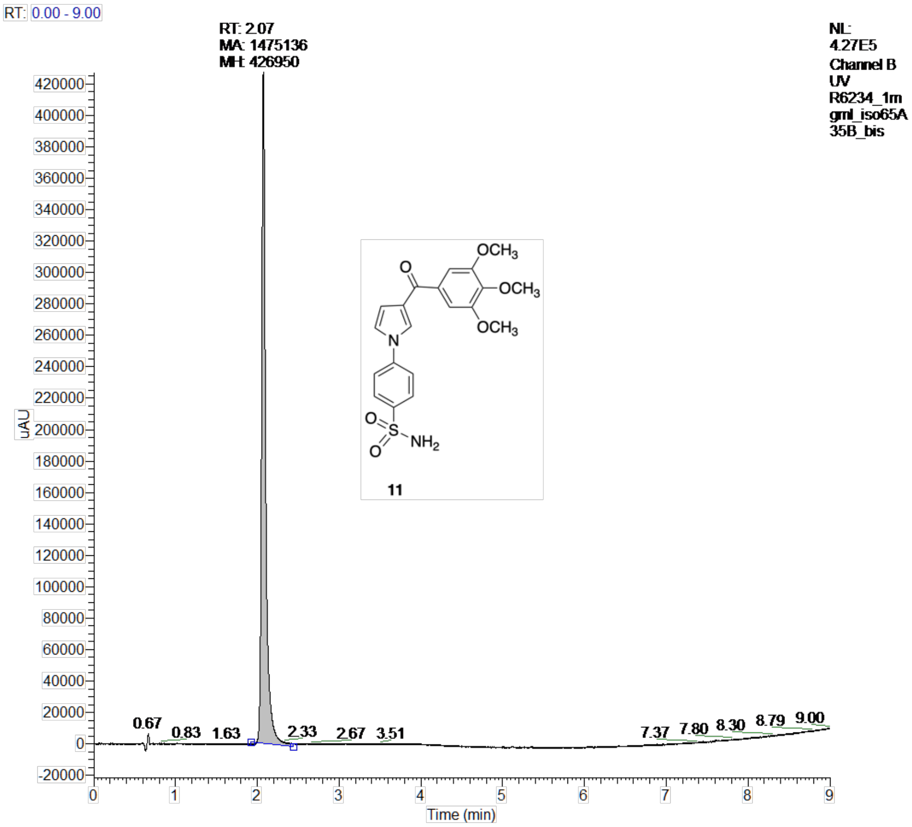

Figure S7. HPLC chromatograms of compounds **11.** Eluents: A) Water + 0,1% TFA, B) ACN + 0,1% TFA. Gradient elution: 0-3 min A/B 65/35; 8 min A/B 10/90; 10 min A/B 10/90.

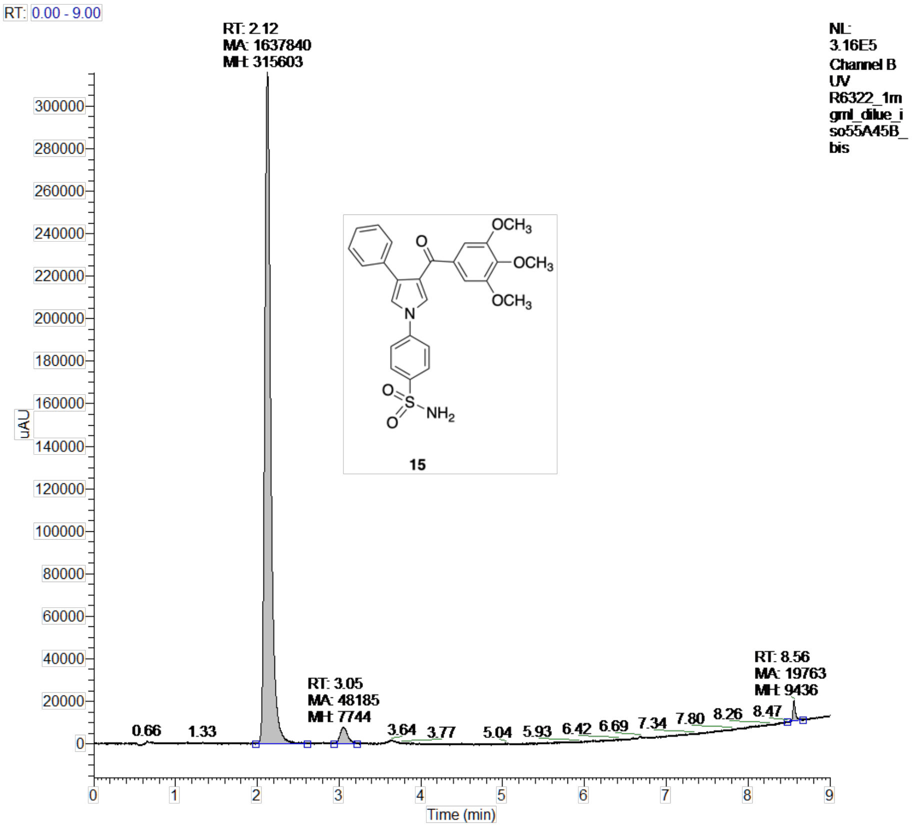

Figure S8. HPLC chromatograms of compounds **15.** Eluents: A) Water + 0,1% TFA, B) ACN + 0,1% TFA. Gradient elution: 0-3 min A/B 55/45; 8 min A/B 10/90; 10 min A/B 10/90.

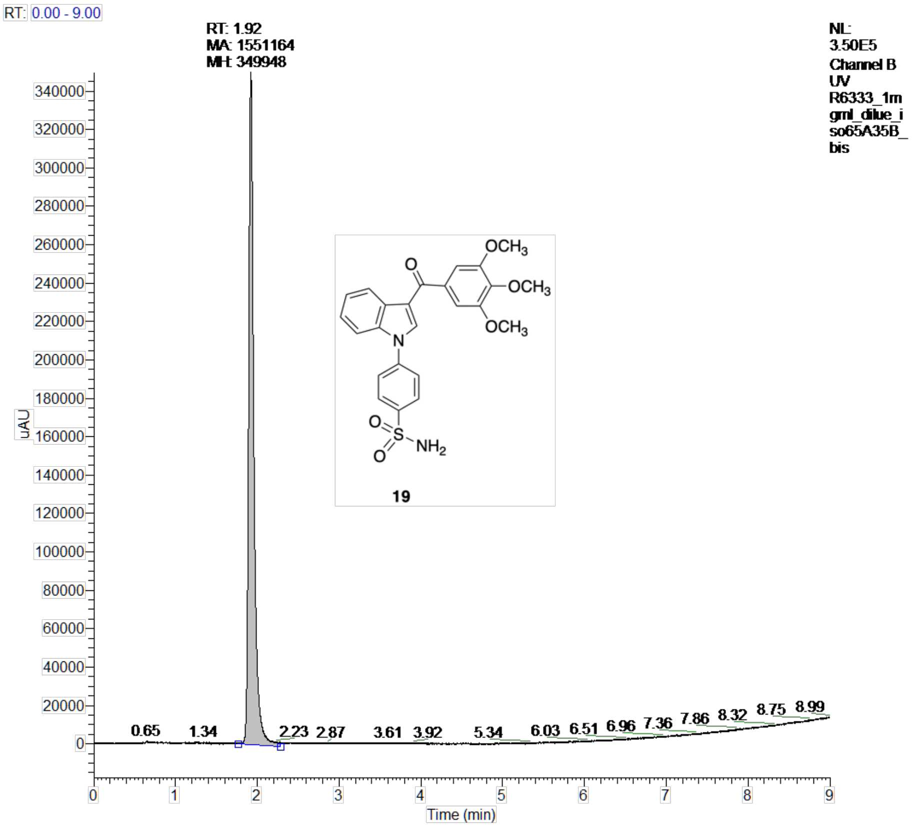

Figure S9. HPLC chromatograms of compounds **19.** Eluents: A) Water + 0,1% TFA, B) ACN + 0,1% TFA. Gradient elution: 0-3 min A/B 55/45; 8 min A/B 10/90; 10 min A/B 10/90.

Table S3. Relative area (%) recorded at 254 nm for compounds **11**, **15** and **19**.

**Compd**	**254 nm Rel. Area (%)**
**11**	>99.99
**15**	96.02
**19**	>99.99

References of SI

(1S) Kopecka, J.; Salzano, G.; Campia, I.; Lusa, S.; Ghigo, D.; De Rosa, G.; Riganti, C. Insights in the chemical components of liposomes responsible for P-glycoprotein inhibition. *Nanomedicine* **2014**, *10*, 77-87.
